# Two-dimensional to three-dimensional knee reconstruction from radiographs and fluoroscopy: A systematic review of methods and accuracy (1995–2025)

**DOI:** 10.1371/journal.pone.0356987

**Published:** 2026-08-26

**Authors:** Yusif Gurbanli, Jaron Mohammed, Francesco Travascio

**Affiliations:** 1 Department of Mechanical and Aerospace Engineering, University of Miami, Coral Gables, Florida, United States of America; 2 Department of Orthopaedic Surgery, University of Miami, Miami, Florida, United States of America; 3 Max Biedermann Institute for Biomechanics at Mount Sinai Medical Center, Miami Beach, Florida, United States of America; Yarmouk University, JORDAN

## Abstract

Three-dimensional reconstruction of the knee plays an important role in orthopedic surgery and clinical decision-making, enabling precise implant planning, evaluation of joint mechanics, and personalized treatment. Computed tomography remains the reference standard for generating 3D bone models, but its use is limited by high radiation dose, cost, and restricted accessibility. This has driven growing interest in reconstructing knee anatomy from two-dimensional radiographs or fluoroscopy as a safer and more widely available alternative. However, the performance of these approaches has not been systematically compared to support clinical translation. This systematic review evaluated methods for 3D knee reconstruction from 2D imaging and compared their accuracy, imaging requirements, computational performance, and clinical applicability. A comprehensive search of seven scientific databases and grey literature sources identified 28 eligible studies spanning statistical shape modeling, deep learning, model-based registration, and hybrid methods. Data extracted included input modality, number of views, validation design, dataset size, accuracy relative to volumetric ground truth, and processing time. Due to variability in reporting and evaluation protocols, a narrative synthesis was performed. Accuracy has improved substantially over three decades, with recent approaches achieving approximately 0.5–1.0 mm root-mean-square error and processing times ranging from under one second to several minutes. Increasing the number of projection views improved reconstruction performance, although clinically acceptable accuracy was often achievable with two orthogonal radiographs. Key limitations included small validation cohorts, limited pathological representation, heterogeneous accuracy metrics, and a lack of public benchmark datasets. Our findings indicate that 3D reconstruction from radiography can achieve accuracy potentially suitable for surgical planning, intraoperative guidance, and longitudinal monitoring, but this is tempered by small validation cohorts, limited pathological representation, and frequent reliance on simulated or digitally reconstructed radiograph (DRR) validation. Future progress requires larger multi-center evaluations, standardized reporting, uncertainty quantification, and integration with clinical workflow systems. With these advances, radiograph-based reconstruction has strong potential to serve as a practical alternative to CT in defined orthopedic applications.

## Introduction

Three-dimensional anatomical reconstruction of the knee joint represents a cornerstone technology in modern orthopedic practice, enabling precise surgical planning, patient-specific implant design, and quantitative biomechanical analysis (Valenti et al., 2016) [1]. While computed tomography (CT) remains the gold standard for skeletal imaging with sub-millimeter resolution (0.2–0.3 mm) [2], its routine application faces significant barriers including radiation exposure (2–10 mSv effective dose), limited accessibility in resource-constrained settings, and substantial costs that challenge healthcare systems globally (Melhem et al., 2016) [3]. Furthermore, CT imaging is typically performed in a non-weight-bearing supine position, whereas X-ray methods can capture patient anatomy in a functional, load-bearing posture [4, 5]. Plain radiographs and fluoroscopy also deliver markedly lower radiation doses for lower limbs (0.1–0.5 mSv) and achieve near-universal availability. While radiographs offer static projections, fluoroscopy additionally provides real-time imaging capabilities essential for dynamic assessment [4, 5]. However, both fundamentally lack the depth information required.

The clinical significance of accurate 3D knee models extends across the continuum of orthopedic care. In preoperative planning for total knee arthroplasty, three-dimensional models facilitate optimal component sizing, alignment optimization, and prediction of bone resection volumes, with studies demonstrating improved postoperative mechanical alignment and reduced outliers when 3D planning is employed (Smoger et al., 2015) [6]. For robotic-assisted surgery, patient-specific 3D models serve as the foundation for haptic boundaries and automated bone preparation, requiring submillimeter accuracy to prevent inadvertent soft tissue injury or suboptimal implant positioning [7]. In kinematic analysis, dynamic 3D reconstruction from fluoroscopy enables non-invasive measurement of in-vivo joint motion patterns, revealing pathological biomechanics alterations in ligament deficiency, meniscal tears, and early osteoarthritis that static imaging cannot capture (Li et al., 2008) [8].

Reconstructing 3D knee geometry from 2D projections represents an ill-posed inverse problem, as infinite 3D configurations can produce identical 2D images (Markelj et al., 2012) [9]. Over three decades, diverse computational strategies have emerged to address this fundamental challenge, from early manual approaches to modern artificial intelligence systems, each incorporating different constraints and prior knowledge to resolve geometric ambiguity. While technological progress has been substantial, with reported accuracies improving from 5–10 mm to sub-millimeter precision, the field lacks comprehensive synthesis comparing these heterogeneous approaches under standardized evaluation criteria for knees in particular [10, 11].

Despite technological advances, current approaches remain constrained by fundamental limitations. Dependence on CT for ground-truth validation creates a circular challenge: methods designed to eliminate CT requirements must be validated against CT, limiting assessment of true clinical performance. The absence of standardized benchmarks impedes objective comparison, studies employ heterogeneous datasets, inconsistent metrics, and varying definitions of accuracy, preventing meta-analysis and evidence-based clinical guidelines [12]. These challenges are not unique to the knee and mirror issues reported in 2D-to-3D reconstruction of other anatomical regions [12]. Small validation cohorts (typically n < 50) limit statistical power and generalizability, while the predominance of healthy subjects fails to address the pathological anatomy encountered in clinical practice where deformity, osteophytes, and bone loss challenge reconstruction algorithms [13]. Furthermore, regulatory pathways for software-as-medical-device remain unclear, and reimbursement mechanisms are absent, creating barriers to clinical adoption despite technical feasibility [14].

Over the past three decades, publications on 2D-to-3D knee reconstruction have increased exponentially, reflecting the growing demand for alternatives to CT-based imaging. However, this rapid progress has resulted in a fragmented landscape of techniques, ranging from statistical shape modeling and model-based registration to deep learning and hybrid approaches, each evaluated using different datasets, imaging geometries, and accuracy metrics. The lack of standardized validation protocols and direct comparisons between methods makes it difficult to determine which approaches are best suited for specific clinical applications. Therefore, a systematic synthesis is critically needed to integrate these diverse methodologies, identify optimal strategies for clinical implementation, and provide an evidence framework that can guide regulatory evaluation and healthcare adoption.

In response to this need, the present systematic review aims to consolidate three decades of research on 2D-to-3D knee reconstruction to evaluate the strategies proposed for converting radiographic or fluoroscopic images into 3D models. Specifically, it examines their input requirements, computational demands, accuracy relative to CT-based ground truth, and reported strengths and limitations. By systematically comparing these approaches, this review seeks to clarify their relative technical readiness, highlight remaining gaps, and outline priorities for future development and clinical translation.

## Methods

### Protocol and reporting standard

This systematic review was conducted in accordance with the Preferred Reporting Items for Systematic Reviews and Meta-Analyses (PRISMA) 2020 statement [15]. The review protocol was developed a priori following a standardized operating procedure document that specified all methodological decisions, search strategies, eligibility criteria, data extraction procedures, and quality assessment methods [16]. The review protocol was not prospectively registered in PROSPERO. We acknowledge this as a limitation: prospective registration improves transparency and reduces the risk of selective reporting, and an iterative search does not preclude registration. To mitigate this, the a priori operating-procedure document, all search strings and dates, the eligibility criteria, and all protocol deviations were documented internally and are reported here in full. The PRISMA 2020 checklist was completed to ensure comprehensive reporting of all required elements [17].

### Databases and search strategy

A comprehensive systematic search was conducted across multiple electronic databases without date restrictions to capture the full evolution of 3D knee reconstruction methodologies. The following databases were searched from inception through November 20, 2025: PubMed/MEDLINE (1966-present), Embase (1947-present), Scopus (1970-present), Web of Science Core Collection (1900-present), IEEE Xplore (1988-present), arXiv preprint repository (1991-present), and Google Scholar (first 500 results per query variant). Grey literature sources included ProQuest Dissertations & Theses Global, conference proceedings from relevant societies (MICCAI, CVPR, IPMI, ISBI, SPIE Medical Imaging), and ClinicalTrials.gov for registered trials involving reconstruction technologies.

The primary search strategy was developed for PubMed using a combination of Medical Subject Headings (MeSH) and text words:

(“knee”[MeSH Terms] OR knee[tiab] OR tibiofemoral[tiab]) AND
(“reconstruction, three-dimensional”[MeSH Terms] OR “3D reconstruction”[tiab] OR
“three dimensional reconstruction”[tiab] OR “3-D reconstruction”[tiab]) AND
(x-ray*[tiab] OR radiograph*[tiab] OR fluoroscop*[tiab] OR “single-plane”[tiab] OR
“bi-planar”[tiab] OR “2D-3D”[tiab] OR “statistical shape model”[tiab] OR
“model-based registration”[tiab] OR “shape-from-silhouette”[tiab] OR
“implicit neural representation”[tiab])

This strategy was adapted for each database according to their specific syntax requirements without language restrictions: IEEE Xplore, Scopus, and Web of Science searches mapped [tiab] to TITLE-ABS-KEY or TOPIC fields, removed MeSH terms, and adjusted wildcard operators. Backward citation searching examined reference lists of all included studies, while forward citation searching using “Cited by” functions in Google Scholar, Scopus, and Web of Science identified papers citing seminal works in the field. The last search update was performed on November 20, 2025, with all search strings, dates, and result counts documented in a search log. We confirm that all searches were completed immediately prior to initial submission and that no eligible study published before the submission date was knowingly omitted; the multilingual search added during revision did not identify additional English-language studies beyond those already captured.For arXiv and Google Scholar, which do not support the full Boolean and field-tag syntax of bibliographic databases, the search was decomposed into a fixed, ordered set of sub-queries executed in the following sequence: (1) “2D 3D knee reconstruction radiograph”; (2) “biplanar radiograph 3D bone reconstruction knee”; (3) “statistical shape model knee X-ray reconstruction”; (4) “deep learning 2D to 3D bone reconstruction knee”; (5) “fluoroscopy 3D knee reconstruction”; and (6) “EOS biplanar 3D reconstruction femur tibia”, each combined with the synonyms “projection geometry,” “biplanar,” “calibration,” and “knee reconstruction radiograph.” For Google Scholar the first 500 results of each sub-query were screened in relevance order (the default ranking), without date restriction; each sub-query was run on the same date and its result count logged. Records retrieved from Google Scholar and arXiv were exported and de-duplicated against the database and register results in the reference manager using DOI and title matching before screening, so that no record was screened or counted twice across sources.

### Eligibility criteria

Studies were included if they involved human knees from in-vivo subjects, cadaveric specimens, or anthropomorphic phantoms, described computational strategies for reconstructing three-dimensional knee models from two-dimensional X-ray radiographs or fluoroscopy images; and reported quantitative reconstruction accuracy against a volumetric reference standard. Acceptable reference standards included CT (preferred), CT-derived digitally reconstructed radiographs (DRRs), MRI-derived 3D models, or clinically validated 3D reconstructions from low-dose biplanar systems (e.g., EOS), Additionally, studies needed to be published and have full-text availability through institutional access or open repositories. For instance, one early study (Laporte et al., 2003) that fit all the other criteria is referenced in the narrative for historical context; however, it was excluded from the quantitative synthesis because the full text was not accessible through institutional subscription. Studies using DRR-only validation were included but tagged separately for further analysis.

Exclusion criteria encompassed studies exclusively reconstructing hip or ankle joints without extractable knee-specific results, proximal femur studies without information on distal femur or tibia, validation using only magnetic resonance imaging without any volumetric bone reference model, and EOS imaging studies that reported only two-dimensional measurements without three-dimensional model reconstruction. Studies were also excluded if full texts were inaccessible after institutional access attempts and interlibrary loan requests, if they consisted of non-peer-reviewed white papers, marketing materials, or patent applications, if they reconstructed only implant components without native anatomical structures, or if they were preprint versions when a peer-reviewed publication of the same work existed. Mixed-joint papers were included only when knee-specific accuracy metrics could be extracted independently.

### Study selection and screening

Study selection followed a two-stage screening process implemented using Rayyan systematic review software [18]. In the first stage, titles and abstracts were screened against the eligibility criteria. All records flagged as potentially relevant or uncertain proceeded to full-text assessment. In the second stage, full texts were retrieved and evaluated in detail, with specific exclusion reasons documented for each excluded study. Two independent reviewers conducted screening at both stages, with disagreements resolved through discussion. Borderline cases and persistent disagreements were compiled into a flag list for adjudication by the principal investigator, including cases of mixed-joint studies with unclear knee extractability, ambiguous ground-truth modalities, or uncertain three-dimensional reconstruction outputs. PRISMA flow diagram counts were logged at each stage, tracking identification from each database, duplicate removal, screening decisions, and final inclusion numbers with exclusion reasons categorized.

### Data extraction and coding

Data extraction utilized a structured comma-separated values (CSV) schema with predefined fields developed through pilot testing on ten studies. The schema captured study identifiers including DOI, FirstAuthorYear, venue, and peer-review status. Technical specifications were comprehensively documented, with AlgorithmFamily categorized as SSM (Statistical Shape Model), model-based registration, DL-CNN (Deep Learning Convolutional Neural Network), DL-implicit/NeRF/SDF (Neural Radiance Fields/Signed Distance Functions), silhouette/contour, or hybrid approaches. CoreTechnique provided free-text descriptions of specific implementations, while InputModality distinguished between radiograph and fluoroscopy sources. AcquisitionMode differentiated simultaneous multi-system setups from single-system sequential acquisitions, NumViews recorded the integer count of X-ray views used, and CalibrationKnown documented whether calibration parameters were known, estimated, or unknown.

Two reviewers independently extracted data using this schema; discrepancies were resolved by consensus, with a third reviewer consulted when agreement could not be reached. When required data were missing or unclear, values were extracted from supplementary materials, figures, or appendices; study authors were not routinely contacted for additional information.

For accuracy metrics, a hierarchical prioritization was employed with mean surface distance (MSD) in millimeters as the first metric when available, followed by 95th percentile Hausdorff distance (HD95), Dice similarity coefficient, and landmark localization error. All reported metrics were captured in an AdditionalMetrics field with units specified. Root Mean Square Error (RMSE) values were extracted when reported as the primary accuracy measure. Validation characteristics included ground-truth modality (CT, MRI, DRR or EOS-based 3D reconstruction), cohort size and type (cadaveric/in-vivo/phantom), and pathology representation. Clinical translation indicators covered regulatory readiness levels ranging from none through prototype, preclinical, and clinical pilot stages to regulatory mentions, along with availability of public datasets or code and specific clinical applications demonstrated.

Acquisition mode distinctions were explicitly defined: simultaneous multi-system referred to EOS or custom rigs capturing orthogonal views in one synchronized event, while single-system sequential encompassed all other configurations including C-arm repositioning or sequential standing radiographs.

### Data synthesis

Given the heterogeneity in reconstruction methods, accuracy metrics, validation approaches, and reporting standards, a narrative synthesis approach was employed rather than meta-analysis. The synthesis was structured to trace methodological evolution chronologically from early photogrammetric approaches through statistical shape models to modern deep learning implementations, identifying key technological transitions and breakthrough innovations. Studies were grouped by their primary algorithmic approach, including SSM, model-based registration, and deep learning methodologies, to enable within-category comparisons and identify performance patterns specific to each methodology.

Further stratification by acquisition mode examined the impact of imaging geometry on reconstruction accuracy, distinguishing between simultaneous versus sequential acquisitions and analyzing the effect of varying numbers of views. Studies using alternating bi-plane fluoroscopy were analyzed separately from those using synchronized systems. Studies validated using only DRRs were analyzed in a separate subsection to assess potential overestimation of real-world performance compared to those validated against actual CT scans. The synthesis emphasized clinically relevant accuracy thresholds, typically ≤ 2 mm for TKA applications, computational feasibility for point-of-care deployment, and evidence of progression toward regulatory approval and clinical implementation. Because accuracy metrics were highly heterogeneous (RMS, mean surface distance, Hausdorff distance, Dice coefficient, and image-based metrics), results were summarized descriptively using ranges and medians rather than pooled quantitative effect estimates. These metrics are not directly interchangeable and should not be compared across studies as if equivalent. Root-mean-square error (RMSE) and root-mean-square (RMS) distance summarize the average squared deviation between corresponding reconstructed and reference points, and are sensitive to large local errors. Point-to-surface (P2S) or mean surface distance measures the average distance from each reconstructed vertex to the nearest point on the reference surface, and does not require explicit point correspondence. The 95th-percentile Hausdorff distance (HD95) reports a near-worst-case boundary deviation and is therefore systematically larger than a mean surface distance for the same reconstruction. All of the preceding are distance metrics expressed in millimetres, whereas the Dice similarity coefficient is a dimensionless overlap ratio (0–1) quantifying volumetric agreement between segmented regions; a high Dice value can coexist with clinically relevant surface deviations, and Dice cannot be converted to a millimetre error. Because the studies reviewed here reported different subsets of these quantities under different validation designs, all reported values are presented with their original metric and units, and no direct numerical ranking across metrics is implied. Findings from dissertations and conference proceedings were planned to be considered separately to evaluate publication bias and identify emerging methods not yet in peer-reviewed literature.

### Risk of bias and reporting bias assessment

**Fig 1:**
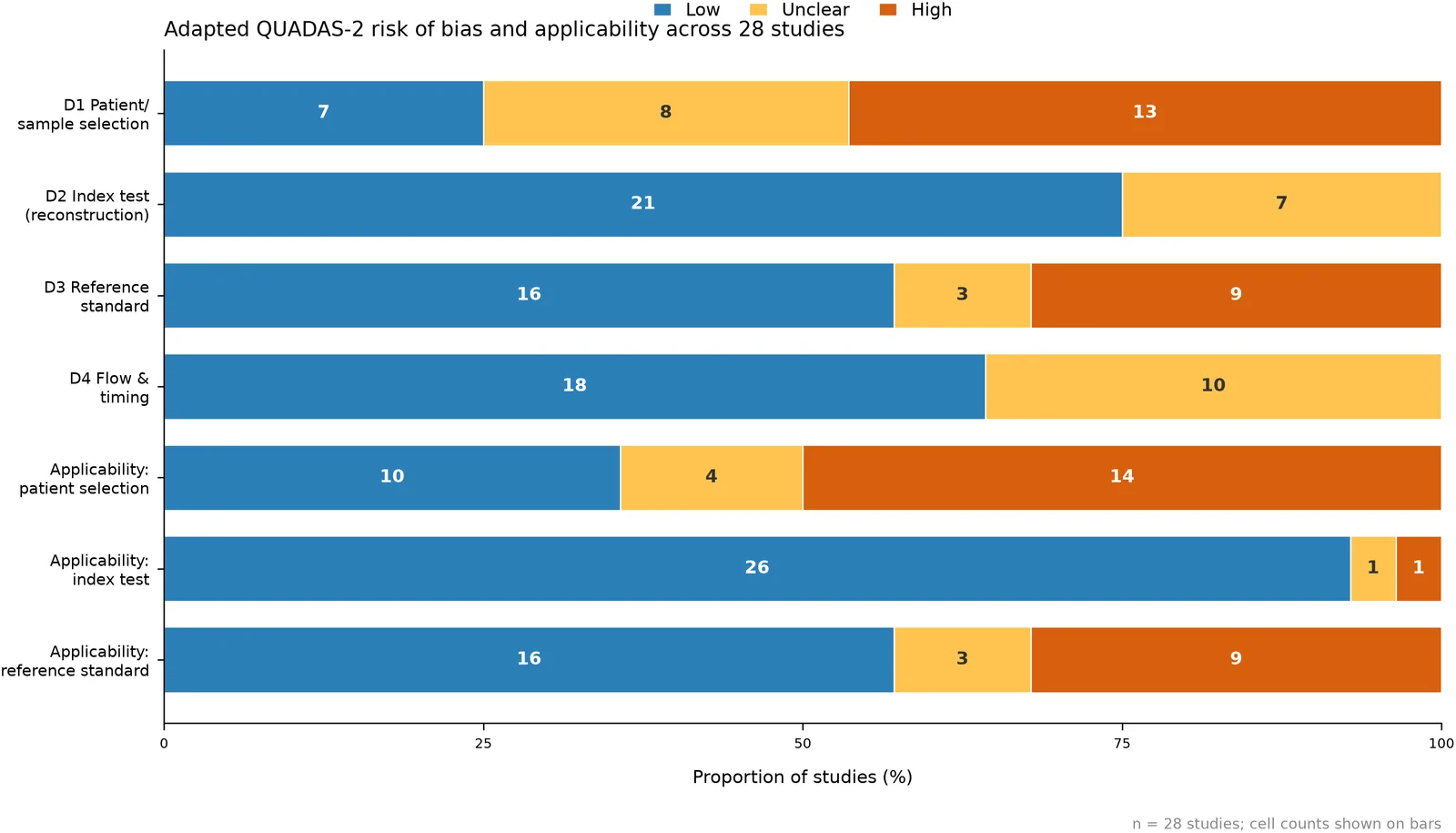
Adapted QUADAS-2 risk-of-bias and applicability judgments across the 28 included studies. Bars show the proportion of studies rated Low, Unclear, or High for each domain; cell counts are shown on the bars.

Because the included studies were technical accuracy-validation studies comparing a reconstruction method against a volumetric reference rather than clinical diagnostic-accuracy or intervention studies, established tools for the latter (ROBINS-I, RoB 2, GRADE) were not applicable. We instead applied an adapted QUADAS-2 framework across all 28 studies. The four QUADAS-2 risk-of-bias domains — patient/sample selection (D1), index test (D2, the reconstruction method), reference standard (D3), and flow and timing (D4) — and the three applicability domains were retained, and domain-specific signalling questions were rewritten for the reconstruction context (full framework in S1 Appendix). Key reinterpretations were that validation on digitally reconstructed radiographs (DRRs) rendered from the same CT used as the reference was treated as a high risk of bias for the reference-standard domain (circularity) and for patient-selection applicability, and that non-CT references (MRI, EOS/SterEOS, or image-only metrics) were treated as weaker standards. Each domain was rated Low, High, or Unclear risk against a prespecified rubric. Judgments were based on the primary full text where retrievable (13/28), on the published abstract (11/28), or, for four studies whose full text could not be obtained, on the descriptive data extracted for this review, with those rows flagged for author verification. Domain-level results are summarized in Fig 1 and per-study ratings are reported in Table 3. Two reviewers independently rated each study against the prespecified rubric, and disagreements were resolved by discussion and consensus, with the senior author adjudicating where needed. Certainty of evidence grading (GRADE) was not conducted because no directly comparable outcome measures or randomized comparisons were available.

## Results and discussion

### Overview of included studies

**Fig 2:**
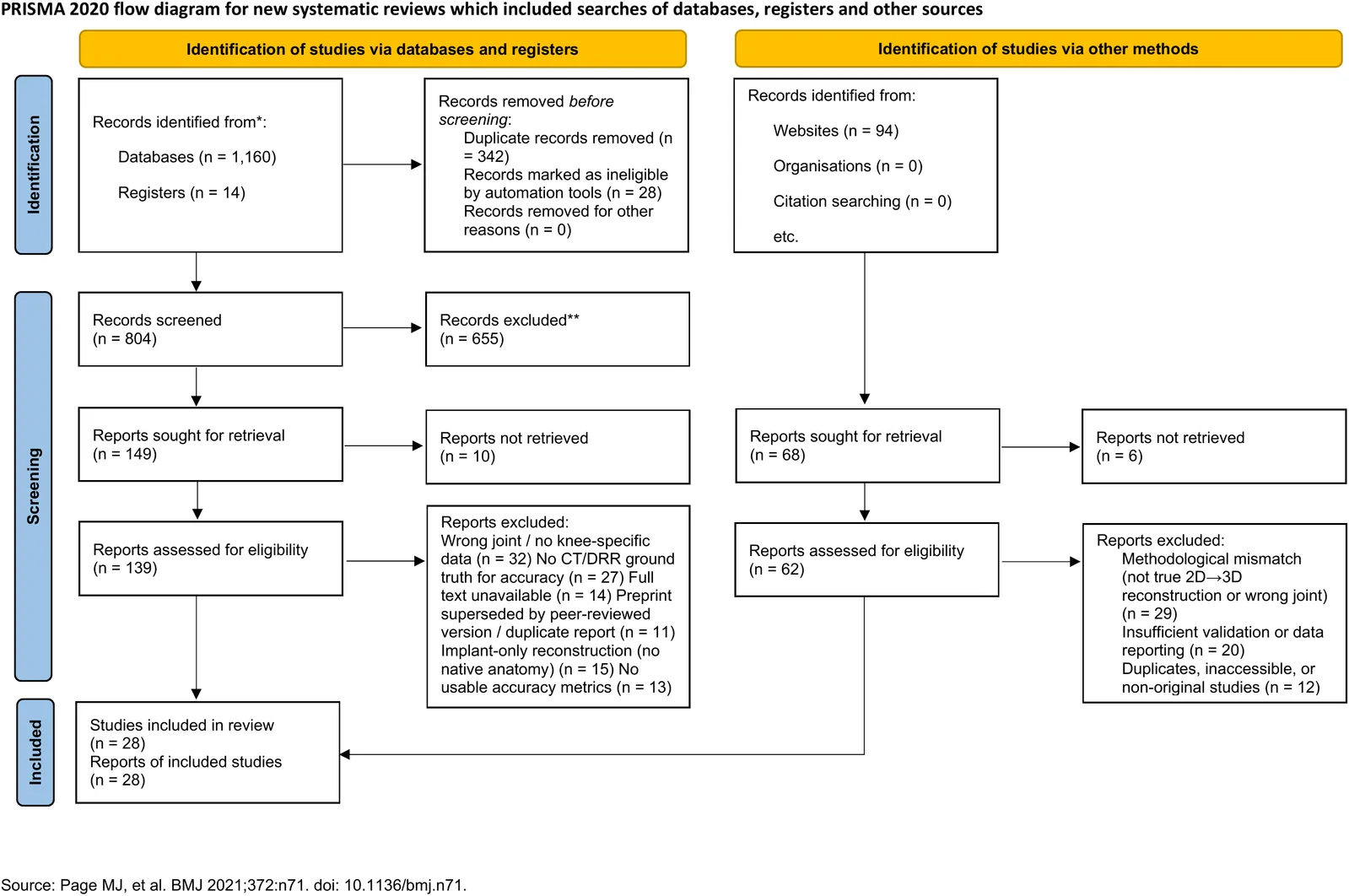
PRISMA 2020 Flow of Study Inclusion. Summary of the screening and selection process for 3D knee reconstruction studies, illustrating record identification, exclusion reasons, and the final inclusion of 28 eligible papers.

The literature search identified 1,268 records in total. Database and register searches yielded 1,174 records (1,160 from databases and 14 from registers); after removal of 342 duplicate records and 28 records excluded by automation tools, 804 records were screened. A further 94 records were identified from other sources (e.g., citation and website searches) and screened separately. Across both streams, 28 studies met all eligibility criteria and were included in the systematic analysis (see Fig 2). These studies represented more than three decades of continuous research advancement from 1995 to 2025, demonstrating sustained academic and clinical interest in developing alternatives to CT-based 3D reconstruction methods; the earliest related work, by Caponetti et al. in 1990, was excluded from the 28 for lack of any quantitative accuracy metrics [19].

**Fig 3:**
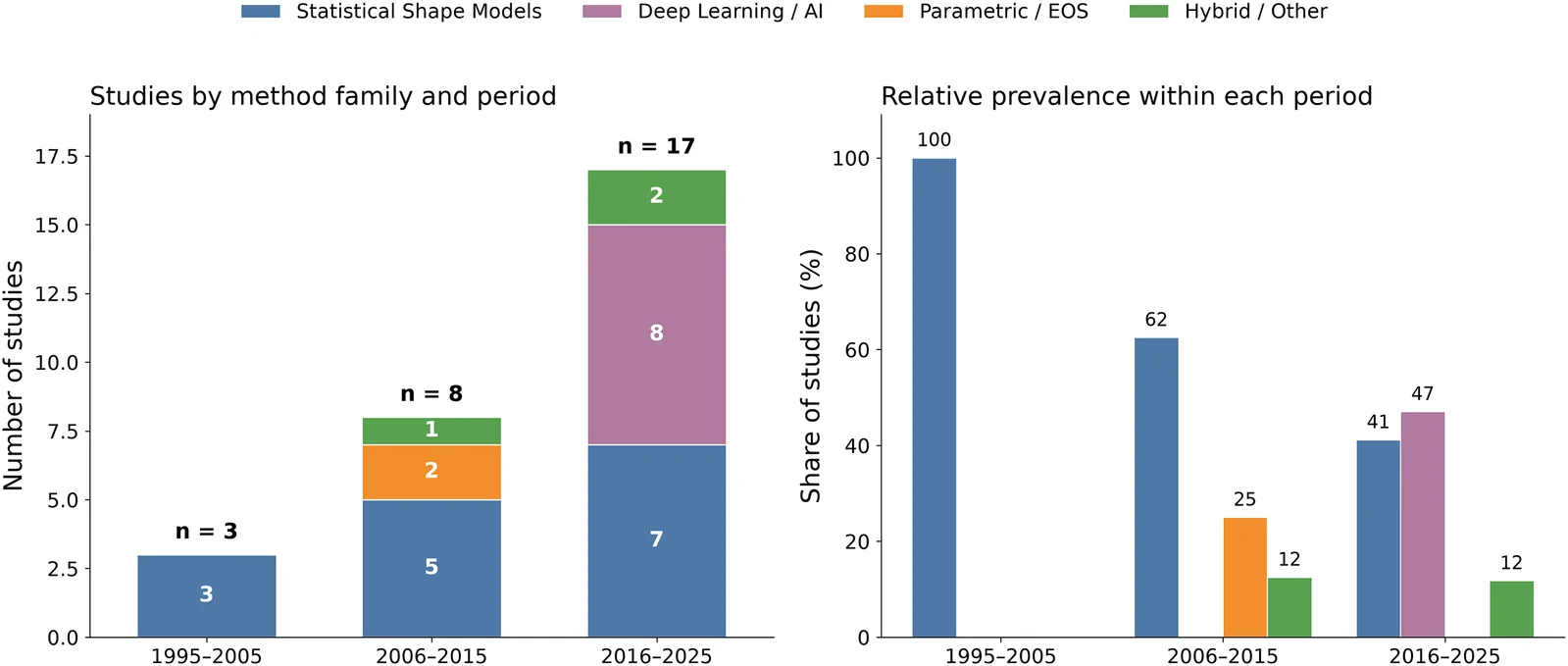
Evolution of 3D bone-reconstruction algorithms across three decades (1995–2025). (A) Absolute number of included studies by method family within each period (1995–2005, 2006–2015, 2016–2025), shown as stacked bars (period totals n = 3, 8, and 17). (B) Relative prevalence of each method family within each period. In the most recent period (2016–2025), deep-learning/AI methods account for 47% (8/17) of studies, statistical shape models for 41% (7/17), and hybrid/other methods for 12% (2/17).

Across the 28 studies, methodological families were distributed as follows: statistical shape models (15 studies, 54%), deep learning/AI-based methods (8 studies, 29%), parametric or EOS-based reconstruction approaches (2 studies, 7%), and hybrid or other geometric/proprietary methods (3 studies, 11%). The temporal distribution of publications showed a clear acceleration in research activity: 3 studies (11%) were published in 1999–2005, 8 studies (29%) in 2006–2015, and 17 studies (61%) in 2016–2025, indicating growing momentum in the field. Geographic distribution demonstrated global research efforts, with European institutions leading contributions, followed by North American and Asian research centers, reflecting the worldwide need for reduced-radiation 3D reconstruction methods. The temporal distribution of method families across these three periods is shown in Fig 3.

The 28 included studies employed heterogeneous validation approaches. Several studies used cadaveric specimens with CT-based ground truth, offering controlled experimental conditions with direct CT correlation [20, 21]. A subset performed in-vivo validation on patient cohorts [11, 22], while others combined anthropomorphic phantoms, simulated DRR-based data, and clinical cases [23, 24].

Study populations varied considerably in size and characteristics. Early SSM studies typically utilized 10–50 training specimens [25] (Fleute & Lavallée, 1999), contemporary SSM approaches employed 60–152 models [22, 26] (Lu et al., 2021; Tsai et al., 2015), while recent deep learning methods leveraged 100s to 1000s of training cases. This exponential growth in training dataset sizes directly correlated with improvements in reconstruction accuracy and generalization capabilities.

### Evolution of Reconstruction Methods (1995–2025)

#### 1995–2005.

Work in this period established SSM as a viable approach for 2D-to-3D reconstruction of knee bones. Fleute and Lavallée (1999) [25] pioneered SSM application to orthopedic knee reconstruction, achieving 0.99 mm root mean square (RMS) point-to-surface (P2S) distance for distal femur reconstruction using merely two orthogonal views. This seminal work demonstrated feasibility despite limited training data and computational resources. This whole wave of papers was heavily inspired by Cootes et al. 1995 study on active shape models [27].

Laporte et al. (2003) [28] advanced biplanar reconstruction methodology by combining 2D and 3D contours for distal femur reconstruction using a small training set. The study is referenced here for historical context, but its quantitative accuracy metrics could not be independently verified from the full text and were therefore not included in the pooled analysis. Other early work, such as Mahfouz et al. (2003) [29], investigated robust multi-component registration techniques for lower-limb anatomy. Though showing the trend of moving from CTs to radiographs/fluoroscopy, this study did not meet the predefined eligibility criteria for this review (focused more on the implant registration rather than bone reconstruction) and was therefore not included in the 28-study dataset. During this period, Tang and Ellis (2005) [30] introduced a hybrid atlas approach combining global and local shape models, reporting errors of approximately 2 mm, as shown on Table 1. These results created baseline accuracy targets and motivated further automation. In addition, we identified a 2005 Master’s thesis by Hung-Chun Lee (written in Chinese), which used a model-based 2D–3D reconstruction approach combining Active Shape Models (ASM), Iterative Closest Point (ICP), and Radial Basis Function (RBF) to reconstruct 3D knee anatomy from biplanar X-rays. Although the study did not report RMSE in millimeters, voxel-based error estimates suggested a 3D reconstruction accuracy of approximately 5 voxels [31].

**Table 1:** Technical characteristics of the 28 included 2D-to-3D knee reconstruction methods: bone/region, descriptive algorithm label, assigned method family (four-family scheme: statistical shape model, deep learning/AI, parametric/EOS-based, or hybrid/other), core technique, input modality, and number of views.

Study	Bone/ region	Algorithm family	Assigned family	Core technique	Input modality	No. of views
Fleute & Lavallée, 1999 [25]	Distal femur	Statistical Shape Model (SSM)	SSM	Non-rigid 3D/2D registration via generalized ICP with contour matching	Calibrated X-rays (C-arm)	2
Baka et al., 2011 [21]	Distal femur	Statistical Shape Model (SSM)	SSM	Edge-based 3D–2D registration + automatic Canny edge selection, PCA model deformation	Biplane fluoroscopy (stereo X-ray)	2
Baka et al., 2014 [33]	Femur & Tibia	Statistical Shape Model (SSM)	SSM	Automated SSM + edge-based tracking using Canny detector + numerical optimizer	Biplane fluoroscopy (drop-landing)	2
Chaibi et al., 2012 [44]	Femur & Tibia	Parametric Statistical Model	Parametric	Simplified Parametric Model + statistical inference (linear regression + MLS deformation)	Biplanar EOS radiographs	2
Quijano et al., 2013 [45]	Femur & Tibia	Parametric Statistical Model	Parametric	Parametric model + multi-linear regression on descriptive parameters + kriging refinement	Biplanar calibrated radiographs (EOS)	2
Gajny et al., 2022 [39]	Femur & Tibia	Statistical Shape Model + Contour Matching	SSM	Quasi-automated 3D reconstruction via SSM and contour alignment with reduced manual input	Low-dose biplanar radiographs (EOS)	2
Fernandes et al., 2023 [10]	Femur & Tibia	Artificial Intelligence (Deep Learning)	Deep learning	AI algorithm converting 2D radiographs to 3D models (unknown architecture)	AP + Lateral radiographs (biplanar)	2
Gothard & Anton, 2025 [46]	Tibia (TKA)	Geometric Model Deformation	Hybrid	Full Ellipse Scaling and Shifting (FESS) method on generic TKA tibia model	Biplanar radiographs	2
Ha et al., 2024 [42]	Femur	Deep Learning + SSM Hybrid	Deep learning	Deep transfer learning network predicting SSM deformation parameters from single X-ray	Single X-ray (AP or unique pose)	1
Kasten et al., 2020 [35]	Femur & Tibia	Deep CNN	Deep learning	End-to-end 3D segmentation and reconstruction from paired AP/Lateral X-rays (no SSM)	Biplanar X-ray images (AP + Lat)	2
Tang & Ellis, 2005 [30]	Proximal & Distal femur	Statistical Shape Model (Hybrid Atlas)	SSM	Hybrid PDM using in-sphere global + local parameterization, intensity-based registration	Simulated & fluoroscopic X-rays	2–4
Tsai et al., 2015 [26]	Distal/Proximal Tibia	Statistical Shape Model (Linear PCA)	SSM	PCA on 152 CT knee models for 3D reconstruction from biplane fluoroscopy	Dual fluoroscopy (biplanar)	2
Li et al., 2014 [34]	Femur & Tibia	Statistical Shape Model + Dual Fluoroscopy Integration	SSM	DFIS + SSM for in vivo kinematics prediction	Biplane fluoroscopy (treadmill walking)	2
Lu et al., 2021 [22]	Femur & Tibia	Statistical Shape Model (CT-based)	SSM	Two-phase optimization for asynchronous fluoroscopy reconstruction	Alternating bi-plane fluoroscopy (1–3 pairs)	2–6
Lu et al., 2023 [47]	Femur & Tibia	SSM + AIMT (interpolation model tracking)	SSM	Alternating interpolation-based model tracking with SSM database	Alternating bi-plane fluoroscopy (1–3 pairs)	2–6
Wu & Mahfouz, 2021 [41]	Femur & Tibia	Nonlinear SSM (Kernel PCA)	SSM	Single-plane fluoroscopy + hybrid intensity & feature energy optimization	Single-plane fluoroscopic sequence (deep knee bending)	1
Factor et al., 2024 [11]	Femur & Tibia (TKA patients)	Deep Learning (AI Model – RSIP XPlan.ai)	Deep learning	CNN-based 2D → 3D reconstruction from X-rays	AP + Lateral radiographs	2
Corona-Figueroa et al., 2022 [24]	Knee (CT reconstruction task)	Deep Learning (Neural Radiance Field – NeRF)	Deep learning	Generative Radiance Fields (GRAF variant) for CT-projection synthesis from X-ray	Single X-ray	1
Roth et al., 2024 [40]	Tibia	Deep Learning (CNN)	Deep learning	Landmark-guided 3D reconstruction from biplanar EOS radiographs	Biplanar EOS (standing radiographs)	2
Babazadeh et al., 2025 [38]	Femur	CNN + Non-rigid Registration	Deep learning	CNN cascade (Pose + Shape + Scale modules) with Moving Least Squares deformation	Biplanar EOS radiographs	2
Cerveri et al., 2017 [23]	Distal femur	Statistical Shape Model (SSM)	SSM	Evolutionary optimization morphing of SSM using DRR similarity	Simulated calibrated X-rays (DRR from CT)	2–5
Valenti et al., 2016 [1]	Femur	Statistical Shape Model (SSM) + Gaussian Mixture Model (GMM)	SSM	2D–3D registration via expectation–conditional maximization using GMM contour correspondence	Fluoroscopy (biplane or single-plane)	6
Karade & Ravi, 2015 [20]	Distal femur	Template-based geometric model	Hybrid	Kohonen self-organizing map for 2D–3D correspondence + Laplacian surface deformation	Simulated & real biplanar radiographs (AP + ML)	2
Shetty et al., 2021 [37]	Femur & Tibia	Proprietary algorithm (XrayTo3D®)	Hybrid	Radiograph-based 3D model generation with shape fitting to X-rays	Standard AP + Lateral radiographs	2
Fotsin et al., 2019 [43]	Knee (femur, tibia, fibula)	Statistical Shape + Pose + Density Model (hybrid PCA)	SSM	PCA-based volumetric SSM + AAM + genetic optimization	Biplanar simulated radiographs	2
Baka et al., 2012 [32]	Distal femur	Statistical Shape Model (SSM)	SSM	Dynamic sequence optimization of 2D–3D registration (minimize edge distance)	Biplane fluoroscopy (drop landing)	2
Massé & Ghate, 2021 [36]	Femur & Tibia	Proprietary AI model (X-Atlas™)	Deep learning	3D reconstruction from standard radiographs for PSI guide design	Standard AP + Lateral radiographs (weight-bearing)	2
Hung-Chun Lee, 2005 [31]	Femur & Tibia	Statistical Shape Model (SSM)	SSM	Active Shape Model (ASM); Radial Basis Function (RBF) for deformable model fitting	Biplanar X-ray radiographs (AP and lateral views)	2

#### 2006–2015.

Over this period of time algorithmic refinement and automation broadened SSM utility. Baka et al. (2011) [21] developed automated edge-based SSM fitting for stereo X-ray reconstruction, achieving 1.68 mm RMS P2S error on biplane fluoroscopy data. Their subsequent work extended this to dynamic sequences (Baka et al., 2012) [32] and established robust kinematic precision (Baka et al., 2014) [33]: 0.48–0.81 mm for translations and 0.69–0.99° for rotations, validated against implanted tantalum bead markers (RMSE of 1.18 mm (femur), 1.56 mm (tibia)).

Tsai et al. (2015) [26] created the most comprehensive SSM database to date, incorporating 152 human CT knee joint models covering femur, tibia, and patella. This landmark study achieved exceptional sub-millimeter accuracy: femur 0.30 ± 0.81 mm, tibia 0.34 ± 0.79 mm, and patella 0.36 ± 0.59 mm, with processing times on the order of a few tens of seconds, setting new standards for clinical feasibility. It’s important to note that these are joint-space/distance differences, not a single global RMS for a bone. Other researchers introduced novel deformation techniques; for instance, Karade and Ravi (2015) [20] proposed a method based on Laplacian surface deformation, achieving 1.5 mm RMS-P2S accuracy with a computation time of less than a minute. Li et al. (2014) [34] also directly compared SSM-based models to CT-based models for kinematic tracking, finding geometric RMS errors of 1.16 mm for the femur and 1.40 mm for the tibia. These data showed that SSMs could satisfy ≤2 mm accuracy with practical runtimes.

#### 2016–2025.

This period has been characterized by the integration of deep learning and hybrid approaches. Kasten et al. (2020) [35] demonstrated end-to-end convolutional neural network reconstruction from biplanar X-rays, achieving Dice scores between 0.85–0.95 while eliminating manual initialization requirements. Processing times (inference) decreased to 0.5 seconds for complete multi-bone reconstruction, representing a 60-fold speed improvement over traditional optimization methods.

Validations of commercial and novel AI algorithms became common. Massé and Ghate (2021) [36] validated the X-Atlas technology, achieving landmark accuracy below 0.87 mm (femur) and 1.28 mm (tibia) compared to MRI. Shetty et al. (2021) [37] validated the XrayTo3D technology, finding no statistically significant difference in 11 anatomical parameters compared to CT-based models and achieving mean P2S errors of 1.0 mm (distal femur) and 1.1 mm (proximal tibia). Factor et al. (2024) [11] validated the RSIP XPlan.ai algorithm on real-world TKA patient anatomies, achieving global RMSE of 0.93 ± 0.25 mm for the femur and 0.88 ± 0.14 mm for the tibia. Critically, accuracy in clinically relevant regions was even higher, with bony landmark RMSE values of 0.51 ± 0.33 mm (femur) and 0.47 ± 0.17 mm (tibia).

Hybrid approaches also matured; Babazadeh et al. (2025) [38] developed an automatic CNN cascade-based 3D/2D non-rigid registration platform, achieving 0.88 mm RMS P2S accuracy with fully automatic processing in 75 seconds, demonstrating the maturity of AI-driven approaches for clinical deployment. Across these studies, SSMs remained competitive in multi-view, calibrated settings, while deep learning and hybrid methods reduced user burden and increased throughput.

### Image acquisition modalities

Studies employing simultaneous multi-system acquisition, primarily using EOS biplanar imaging systems (n = 5), achieved mean reconstruction accuracy of 0.9–1.6 mm. These systems benefit from synchronized orthogonal image capture, eliminating inter-view motion artifacts. Quijano et al. (2013) exemplified EOS performance, reporting mean shape errors of about 1.5–1.6 mm for the femur and tibia, while more recent EOS-based approaches reported errors close to 1 mm (Gajny et al., 2022; Babazadeh et al., 2025) [38, 39]. Similarly, Roth et al. (2024) [40] utilized biplanar EOS radiographs for a deep-learning pipeline to plan high tibial osteotomies (HTO).

Sequential single-system acquisition, representing the majority of clinical scenarios, overall demonstrated slightly degraded but clinically acceptable accuracy. To address this, Lu et al. (2021) [22] specifically addressed asynchronous fluoroscopy challenges using a two-phase optimization approach, achieving remarkable accuracy despite temporal separation between views.

Single-view reconstruction methods showed expected accuracy degradation, with out-of-plane measurements particularly affected. Wu and Mahfouz (2021) [41] pushed single-view limits using nonlinear kernel PCA-based SSM, achieving 1.19 mm (femur) and 1.15 mm (tibia) RMS accuracy from lateral fluoroscopy alone. Ha et al. (2024) [42] employed deep transfer learning for single X-ray reconstruction, achieving 1.20 mm and 1.08 mm errors for proximal and distal femur respectively. Other novel approaches like MedNeRF (Corona-Figueroa et al., 2022) [24] have also explored reconstructing 3D-aware CT-projections from a single X-ray.

**Fig 4:**
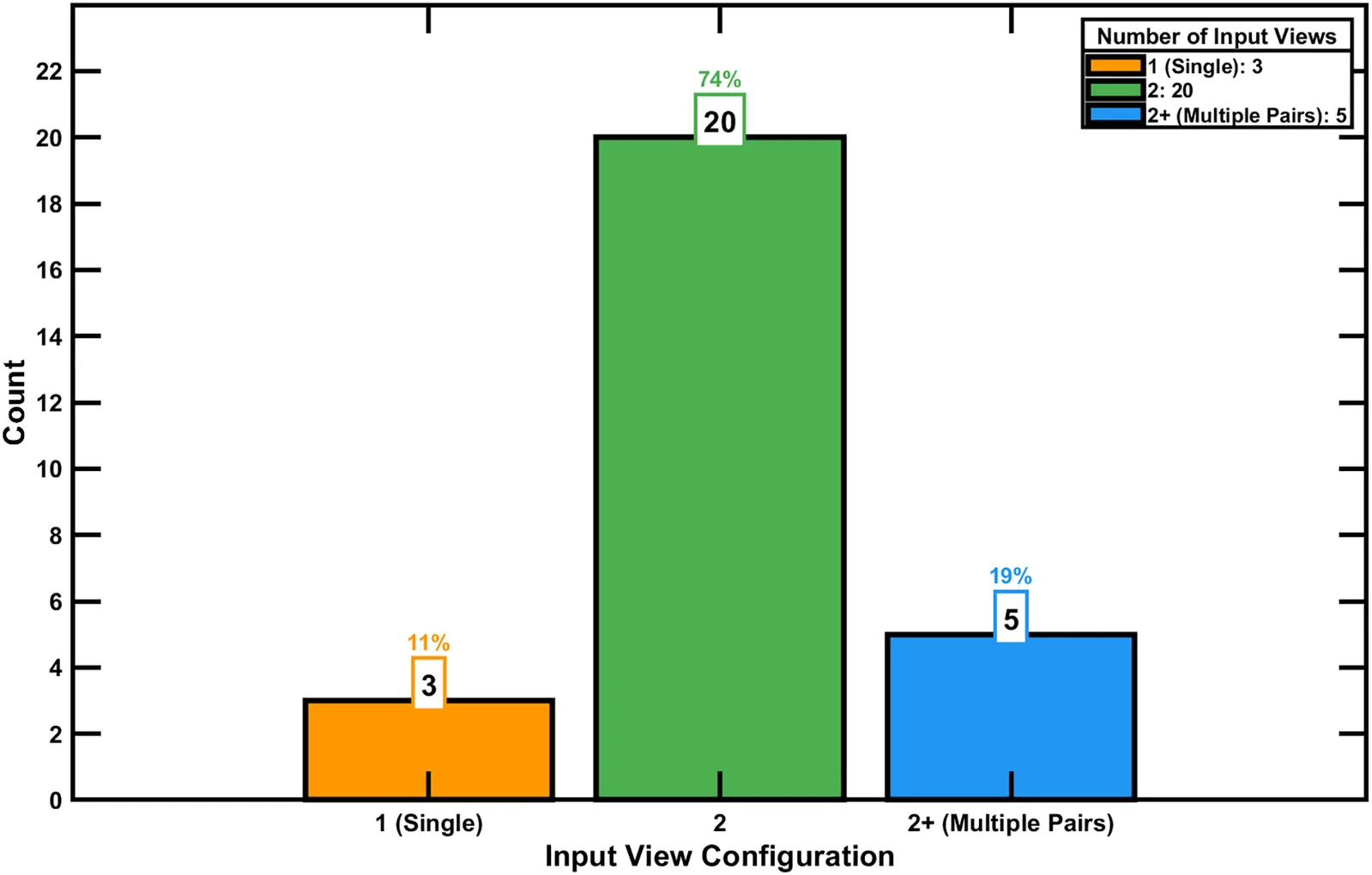
Distribution of Input View Configurations in 2D-to-3D Knee Reconstruction Methods.

Studies utilizing multiple projection angles demonstrated superior accuracy through redundant geometric constraints. Lu et al. (2021) [22] systematically evaluated the impact of image pair quantity on reconstruction accuracy. Using one image pair (two views) achieved 0.81 ± 0.126 mm for the femur and 0.83 ± 0.108 mm for the tibia. Two image pairs (four views) improved accuracy to 0.68 ± 0.088 mm for the femur and 0.73 ± 0.04 mm for the tibia. Three image pairs (six views) yielded the best results at 0.52 ± 0.09 mm for the femur and 0.63 ± 0.085 mm for the tibia (in simulation). No significant improvements were observed beyond three image pairs, suggesting an optimal balance between accuracy and acquisition complexity. This trend was recognized early; Tang and Ellis (2005) [30] noted that using four images (1.61–1.70 mm RMS error) produced better results than three (1.66–1.99 mm) or two (1.73–2.34 mm). The distribution of input view configurations across the included studies is summarised in Fig 4.

### Quantitative performance analysis

#### Shape reconstruction accuracy.

Recent clinically validated radiograph-based 3D reconstruction systems achieved errors on the order of 0.9–1.1 mm (Factor et al., 2024; Shetty et al., 2021), representing an improvement over earlier methods with errors frequently in the 1.4–2.2 mm range [11, 37]. Reported accuracies, the corresponding metric types, and the validation reference used in each study are compiled in Table 2.

**Table 2:** Performance metrics of 2D-to-3D knee reconstruction methods: reported accuracy, accuracy metric type, and stated validation reference. Metrics are not directly comparable across studies (see Methods).

Study	Reported accuracy	Accuracy metric type	Validation reference (as stated)
Fleute & Lavallée, 1999 [25]	0.99-1.33 mm (200 rays vs 10 rays)	Surface distance (mm)	CT
Baka et al., 2011 [21]	0.68 mm (rigid) – 1.68 mm (P2S non-rigid SSM)	P2S/RMS surface	CT
Baka et al., 2014 [33]	1.18 mm (femur), 1.56 mm (tibia)	Surface distance (mm)	CT + implanted tantalum-bead gold standard
Chaibi et al., 2012 [44]	Mean femoral shape accuracy = 1.2 mm vs CT	Surface distance (mm)	CT (EOS setting)
Quijano et al., 2013 [45]	1.55 mm (femur) (2RMS = 3.1 mm), 1.6 (tibia) (2RMS = 3.2 mm)	RMS surface	CT
Gajny et al., 2022 [39]	≈ 1.0 mm (RMSE P2S distance)	P2S/RMS surface	CT
Fernandes et al., 2023 [10]	< 2 mm	Surface distance (mm)	CT + manual calipers
Gothard & Anton, 2025 [46]	1.19 mm (max Hausdorff distance)	Hausdorff	Not clearly specified
Ha et al., 2024 [42]	Proximal = 1.20 mm (P2S RMS); Distal = 1.08 mm (P2S RMS)	P2S/RMS surface	DRR (simulated)
Kasten et al., 2020 [35]	Chamfer distance ≈ 1.29 mm (bones average)	Chamfer	DRR (simulated from CT)
Tang & Ellis, 2005 [30]	1.72-1.95 mm	Surface distance (mm)	DRR (simulated) + cadaver fiducials
Tsai et al., 2015 [26]	Joint-space differences: 0.30 ± 0.81 mm (Femur), 0.34 ± 0.79 mm (Tibia), 0.36 ± 0.59 mm (Patella)	Surface distance (mm)	CT
Li et al., 2014 [34]	1.16 mm (femur) and 1.40 mm (tibia)	Surface distance (mm)	CT
Lu et al., 2021 [22]	0.81/0.83/0.68/0.69 mm	Surface distance (mm)	CT (+ simulation)
Lu et al., 2023 [47]	1 pair: Femur 0.83 ± 0.13, Tibia 0.84 ± 0.11 2 pairs: Femur 0.70 ± 0.09, Tibia 0.71 ± 0.05 3 pairs: Femur 0.66 ± 0.09, Tibia 0.67 ± 0.07	P2S/RMS surface	CT
Wu & Mahfouz, 2021 [41]	1.19 ± 0.36 (femur) and 1.15 ± 0.17 (tibia)	P2S/RMS surface	CT
Factor et al., 2024 [11]	0.93 ± 0.25 (femur) and 0.88 ± 0.14 (tibia)	RMS surface	CT
Corona-Figueroa et al., 2022 [24]	Quantitative improvement over baseline (CT projection metrics, no mm reported)	Image-space metrics	Image-space metrics only
Roth et al., 2024 [40]	Mean Euclidean distances: 1.21 ± 0.38 mm (right), 1.63 ± 0.74 mm (left)	Euclidean landmark	DRR (simulated from CT)
Babazadeh et al., 2025 [38]	0.88 ± 0.29 mm (vs. SterEOS fuzzy gold standard); 2.70 ± 0.39 mm (vs. CT models)	Surface distance (mm)	SterEOS + CT (DRR inputs)
Cerveri et al., 2017 [23]	Median RMS error ≈ 0.74–0.80 mm with 3 DRRs; Hausdorff ≈ 1.5 mm (max < 1.8 mm). Using 2 DRRs ≈ 1.0 mm RMSE/ 2.0 mm Hausdorff.	Hausdorff	DRR (simulated from CT)
Valenti et al., 2016 [1]	absolute mean error: 3–4 mm (median)	Surface distance (mm)	Not clearly specified
Karade & Ravi, 2015 [20]	Mean RP2S (RMS point-to-surface distance): 1.5 mm (simulated), 1.4 mm (real)	P2S/RMS surface	DRR (simulated) + biplanar
Shetty et al., 2021 [37]	mean P2S ≈ 1.0 mm (femur), 1.1 mm (tibia)	P2S/RMS surface	CT
Fotsin et al., 2019 [43]	Femur = 0.72 ± 0.31 mm, Tibia = 0.99 ± 0.30 mm, Fibula = 0.82 ± 0.30 mm	Surface distance (mm)	DRR (simulated from CT)
Baka et al., 2012 [32]	1.48 mm ± 0.41 mm	Surface distance (mm)	CT
Massé & Ghate, 2021 [36]	Mean Absolute Error: ≤ 0.87 ± 0.47 mm (femur), ≤ 1.28 ± 0.72 (tibia)	Mean absolute error	MRI
Hung-Chun Lee, 2005 [31]	Not reported in mm (average segmentation error < 6 pixels; 3D reconstruction error ≈ 5 voxels)	Image-space (px/voxel)	Not clearly specified

Larger SSM training sets were associated with lower surface errors: Tsai et al. (2015) [26], using 152 CT knee models, reported mean surface errors of approximately 0.3–0.4 mm across femur, tibia, and patella (joint spaces), whereas earlier SSM studies with fewer than 50 training cases, such as Baka et al. (2011) [21], reported point-to-surface errors around 1.5 mm. Fotsin et al. (2019) [43], for example, used a dataset of 200 subjects to create a combined shape, pose, and density model, achieving mean RMS errors of 0.72 mm (femur) and 0.99 mm (tibia) against simulated data. Reported generalization depended on anatomy coverage, imaging protocol, and calibration knowledge.

### Kinematic measurement precision

For dynamic tracking applications, kinematic precision emerged as the critical performance metric. The AIMT technique with SSM-reconstructed models (Lu et al., 2023) [47] achieved exceptional precision using two image pairs, with translations showing 0.75–0.79 mm mean absolute difference and rotations demonstrating 0.75–0.89° mean absolute difference. These results matched or exceeded synchronous biplane fluoroscopy benchmarks, with median precision of 0.48–0.81 mm for translations and 0.69–0.99° for rotations reported by Baka et al. (2014) [33]. However, Li et al. (2014) [34] found higher kinematic errors when using SSM models, with RMS errors up to 3.3° for rotation and 2.4 mm for translation, suggesting that model accuracy and registration technique are both critical for kinematic precision. Taken together, kinematic precision depends on both reconstruction accuracy and the tracking pipeline.

### Clinical parameter accuracy

Anatomical measurements critical for surgical planning showed high fidelity across reconstruction methods. Factor et al. (2024) [11] demonstrated that AI-based reconstruction achieved 1.89° deviation for the trans-epicondylar axis compared to a baseline of 1.43°, and 1.78° deviation for the posterior condylar axis against a baseline of 1.71°. Shetty et al. (2021) [37] found no significant difference for 11 anatomical parameters, including mLDFA and MPTA, between X-ray-based 3D models and CT models. These accuracies fall within acceptable ranges for clinical decision-making and surgical planning applications.

### Evolution of computational efficiency

Reconstruction times fell by orders of magnitude across the review period. Manual segmentation in 1995–2000 required hours to days for reconstruction. The semi-automated methods from 2001–2010 reduced processing to 30–60 minutes. Automated SSM methods from 2011–2019 achieved reconstruction in 30 seconds to 5 minutes (Baka et al., 2012; Tsai et al., 2015), while deep learning inference from 2020–2025 has reduced times to 0.5–75 seconds for inference [26, 32]. Modern GPU-accelerated deep learning methods achieve near-real-time processing, with Kasten et al. (2020) [35] reporting 0.5 seconds for complete knee reconstruction and Babazadeh et al. (2025) [38] reporting 75 seconds for their full automated pipeline. Acquisition, calibration, and pre-processing remained the practical bottlenecks in some workflows.

### Parametric and hybrid methods performance

The EOS-based parametric reconstruction methods demonstrated unique advantages for complete lower limb assessment. Chaibi et al. (2012) [44] introduced fast 3D reconstruction using simplified parametric models (SPM) with statistical inferences, achieving initial solution accuracy of 1.2 mm for the mean femoral shape accuracy versus the CT standard.

Gajny et al. (2022) [39] advanced quasi-automated reconstruction significantly, reducing operator dependence while maintaining shape accuracy around 1.0 mm. The method’s repeatability (2SD) was excellent: 0.4 degrees for HKS and 4.8 degrees for femoral torsion, demonstrating robustness for clinical implementation. Gothard and Anton (2025) [46] also introduced a non-iterative, hybrid method (FESS) that relies on elliptical scaling from a single generic model rather than a statistical database, achieving an average Hausdorff distance of 1.19 mm with low computational time. These approaches seek to combine SSM stability with learned features for automation.

#### Methodological quality (risk of bias).

Methodological quality (adapted QUADAS-2). Across the 28 studies, risk of bias was concentrated in the patient/sample-selection and reference-standard domains, while the reconstruction methods themselves (index test) and the analysis flow were generally sound (Fig 1, Table 3). Patient/sample selection (D1) was rated high risk in 13 studies (46%) and unclear in 8 (29%), driven by cadaver-, phantom-, or DRR-only samples, by cohorts restricted to healthy or single-population anatomy, and by very small test sets — several pivotal studies validated on as few as two to five specimens. The corresponding applicability concern for patient selection was high in 14 studies (50%), reflecting how often the images used were not the routine clinical radiographs the methods are ultimately intended for.

**Table 3:** Per-study adapted QUADAS-2 ratings. D1 patient/sample selection; D2 index test (reconstruction method); D3 reference standard; D4 flow and timing; A1-A3 applicability concerns for patient selection, index test, and reference standard. Rating source indicates whether the judgment was based on retrieved full text, the published abstract, or the review’s secondary description (author verification recommended).

Study	Family	D1	D2	D3	D4	A1	A2	A3	Rating source
Fleute & Lavallée, 1999	SSM	High	Unclear	Low	Unclear	High	Low	Low	secondary
Baka et al., 2011	SSM	High	Low	Low	Low	High	Low	Low	abstract
Baka et al., 2014	SSM	Low	Low	Low	Low	Low	Low	Low	full-text
Chaibi et al., 2012	parametric	Unclear	Low	Low	Unclear	High	Low	Low	abstract
Quijano et al., 2013	parametric	Unclear	Low	Low	Unclear	Low	Low	Low	abstract
Gajny et al., 2022	SSM	Low	Low	Low	Low	Low	Low	Low	full-text
Fernandes et al., 2023	DL	High	Unclear	Low	Low	High	Low	Low	abstract
Gothard & Anton, 2025	hybrid	Unclear	Low	Unclear	Unclear	Unclear	Low	Unclear	secondary
Ha et al., 2024	DL	High	Unclear	High	Unclear	High	Low	High	secondary
Kasten et al., 2020	DL	High	Low	High	Low	High	Low	High	full-text
Tang & Ellis, 2005	SSM	High	Low	High	Low	High	Low	High	full-text
Tsai et al., 2015	SSM	High	Low	Low	Low	High	Low	Low	full-text
Li et al., 2014	SSM	High	Low	Low	Low	Low	Low	Low	full-text
Lu et al., 2021	SSM	High	Low	Low	Low	High	Low	Low	full-text
Lu et al., 2023	SSM	High	Low	Low	Low	High	Low	Low	full-text
Wu & Mahfouz, 2021	SSM	High	Low	Low	Low	High	Low	Low	full-text
Factor et al., 2024	DL	Low	Unclear	Low	Low	Low	Low	Low	full-text
Corona-Figueroa et al., 2022	DL	High	Low	High	Unclear	High	High	High	abstract
Roth et al., 2024	DL	Unclear	Low	High	Unclear	High	Low	High	full-text
Babazadeh et al., 2025	DL	Unclear	Low	High	Low	High	Low	High	full-text
Cerveri et al., 2017	SSM	Low	Low	High	Low	Low	Low	High	abstract
Valenti et al., 2016	SSM	Unclear	Low	Low	Unclear	Unclear	Low	Low	abstract
Karade & Ravi, 2015	hybrid	Unclear	Low	Unclear	Unclear	Unclear	Low	Unclear	abstract
Shetty et al., 2021	hybrid	Low	Unclear	Low	Low	Low	Low	Low	full-text
Fotsin et al., 2019	SSM	Low	Low	High	Low	Low	Low	High	abstract
Baka et al., 2012	SSM	High	Low	Low	Low	Low	Low	Low	abstract
Massé & Ghate, 2021	DL	Low	Unclear	High	Low	Low	Low	High	abstract
Hung-Chun Lee, 2005	SSM	Unclear	Unclear	Unclear	Unclear	Unclear	Unclear	Unclear	secondary

The reference standard (D3) was the other dominant concern: 9 studies (32%) were rated high risk and 3 (11%) unclear. The recurring problem was circular validation, in which the 2D inputs were digitally reconstructed radiographs rendered from the same CT volume that also supplied the reference surface, so the reported sub-millimetre errors partly measure internal consistency of the rendering-and-fitting pipeline rather than real-world accuracy. A related concern was reliance on weaker reference standards — MRI-derived models, EOS/SterEOS reconstructions, or image-only CT-projection metrics — rather than independent CT segmentation; Babazadeh et al. (2025), for instance, reported 0.88 mm error against a SterEOS “fuzzy gold standard” but 2.70 mm against true CT models, illustrating how the choice of reference inflates apparent performance.

By contrast, the index test (D2) carried no high-risk ratings; 21 studies (75%) were low risk, with the remaining 7 (25%) unclear almost entirely because the method was a proprietary, black-box commercial system whose internal parameters and train/test separation could not be independently verified. Flow and timing (D4) was low risk in 18 studies (64%) and unclear in the remainder, chiefly where cohort flow or exclusions were incompletely reported; no study showed clear evidence of biased flow. Taken together, the appraisal indicates that the methods in this field are technically well constructed and their reported accuracies are internally credible, but the evidence base for real-world clinical accuracy is limited by how the methods were validated rather than by how they were built: heterogeneous and often circular (DRR-based) validation, non-CT reference standards, and small, pathology-poor cohorts all tend to make reported accuracy an optimistic estimate of clinical performance.

### Strengths and Limitations

#### Reported strengths.

Recurring strengths identified across studies centered on clinical translation readiness. Radiation dose reduction was a primary motivator, with biplanar radiography systems like EOS offering 10-50x lower doses than conventional CT (Quijano et al., 2013) [45]. Equipment compatibility with existing radiographic and fluoroscopic systems was emphasized (Shetty et al., 2021) [37], eliminating the need for specialized hardware investments. Near-real-time processing capability was demonstrated (Kasten et al., 2020) [35], enabling intraoperative applications. Cost-effectiveness showing significant reduction versus CT-based planning was quantified (Fernandes et al., 2023; Shetty et al., 2021) [10, 37], supporting economic feasibility for widespread adoption.

### Limitations

Consistent limitations reported across studies revealed important research gaps. Small validation cohorts were a concern, limiting statistical power and generalizability assessment. Limited pathological diversity affected many studies, which tested only healthy or mildly arthritic knees (Cerveri et al., 2017) [23]. This is a critical gap, as studies have demonstrated that pathological conditions like osteoarthritis (OA) are fundamentally linked to joint shape, with OA knees exhibiting significant shape differences, such as increased bone width and an elevated lateral tibial plateau, compared to healthy controls [48]. Population specificity remained an issue, as most SSMs were trained on single ethnic populations (e.g., a Chinese male population in Lu et al., 2021) [22], potentially limiting global applicability. Uncertainty quantification was often absent, hindering clinical trust and adoption. Studies using digitally reconstructed radiographs tended to overestimate accuracy relative to clinical radiographs. A related and under-appreciated source of heterogeneity is that the reference standards used across studies are not equivalent, so reported accuracies cannot be pooled or compared at face value. CT segmentation is the most rigorous volumetric reference and provides genuine bone-surface ground truth, but MRI-derived models depend on soft-tissue-optimised contrast and segmentation choices that shift the apparent bone boundary; EOS/SterEOS reconstructions are themselves model-based estimates (a “fuzzy” reference rather than an independent gold standard), so agreement with EOS partly measures agreement between two reconstruction models; and digitally reconstructed radiographs (DRRs) generated from the same CT used to build or evaluate the model introduce circularity, because the index test is validated against projections of its own reference. Consequently, a method reporting sub-millimetre error against DRRs or against SterEOS is not directly comparable to one reporting the same error against independent CT segmentation, and the former should be interpreted as an optimistic bound. In this review, studies were tagged by reference-standard type so that accuracy figures are read within, rather than across, validation designs; this distinction is reflected in the adapted QUADAS-2 Reference Standard domain.

Method comparison is complicated by heterogeneous datasets (imaging protocols, anatomical coverage, ground-truth definitions) and inconsistent metrics across studies (surface distance measures such as RMS/MAD/Hausdorff, overlap coefficients such as Dice, and landmark errors), which limits meta-analysis and fair benchmarking. Public clinical datasets remain scarce; only recent works provide broader clinical validation with statistical analysis. Selective reporting bias could not be formally assessed because most studies did not provide complete information regarding dataset composition or negative outcomes.

### Clinical implementation readiness

To reduce subjectivity, we classified methods into three translational-readiness tiers using predefined quantitative criteria specified a priori (Table 4). A method was assigned to a tier only if it satisfied all criteria for that tier; where a method met criteria across tiers, it was assigned to the lower (less-ready) tier. The criteria were: (i) Deployment-candidate – reconstruction accuracy of <1 mm (RMS or mean point-to-surface distance) relative to a volumetric reference, processing time <1 min, a substantially automated pipeline, and validation on real (non-simulated) clinical or cadaveric images; (ii) Near-ready – accuracy of 1–2 mm, processing time <5 min, and at most limited manual interaction; (iii) Research-phase – accuracy >2 mm or processing time >5 min, reliance on extensive manual interaction, or validation limited to simulated/DRR data. These thresholds are pragmatic and derived from reported orthopaedic surgical-planning tolerances rather than from a formal outcome study, and are intended as a transparent, reproducible classification rather than a claim of regulatory or clinical qualification.

**Table 4:** Predefined criteria for the three translational-readiness tiers. A method is assigned to a tier only if it satisfies all criteria for that tier.

Tier	Accuracy (RMS/ mean P2S vs volumetric reference)	Processing time	Automation	Validation data
Deployment-candidate	< 1 mm	< 1 min	Substantially automated	Real clinical or cadaveric images (non-simulated)
Near-ready	1–2 mm	< 5 min	At most limited manual interaction	Real or mixed real/simulated images
Research-phase	> 2 mm	> 5 min	Extensive manual interaction	Simulated/DRR-only validation acceptable

Deployment-candidate methods met all criteria: sub-millimetre accuracy against a non-simulated volumetric reference, processing under one minute, and substantial automation. Factor et al. (2024) [11], using the RSIP XPlan.ai system, meets these criteria on real radiographs. Lu et al. (2021, 2023) [22, 47] report sub-millimetre multi-view SSM accuracy and high automation; because part of the sub-1 mm performance for Lu et al. (2021) was established in simulation, we classify it here only where the accuracy criterion is met against real images and otherwise place it in the near-ready tier. Even for deployment-candidate methods, each was validated in a small cohort and none has undergone the multi-centre, prospective evaluation across diverse pathology that clinical deployment would ultimately require.

Near-ready approaches achieved 1–2 mm accuracy with processing under five minutes and at most limited manual interaction. Kasten et al. (2020) [35], whose end-to-end CNN reports a Chamfer distance of approximately 1.29 mm, falls in this tier rather than the deployment-candidate tier because its reported accuracy exceeds the sub-millimetre threshold. Gajny et al. (2022) [39], with quasi-automated EOS reconstruction, and Ha et al. (2024) [42], using single-view deep transfer learning, also fall here. Massé and Ghate (2021) [36] fits this tier, showing landmark accuracy below 1.28 mm (tibia) and high implant-sizing prediction rates, though it relies on trained operators.

Research-phase methods are characterised by accuracy exceeding 2 mm, processing times over five minutes, extensive manual interaction, or validation only against simulated/DRR references. Applying the drop-to-lower-tier rule, Babazadeh et al. (2025) [38] is placed here: although it reports 0.88 mm against a SterEOS reference, its error against true CT reaches 2.70 mm, which governs classification. This tier also includes single-view methods without deep learning, approaches requiring extensive manual interaction, and methods lacking validation on pathological models. These approaches require substantial further development before clinical consideration.

## Conclusion

This review indicates that three-dimensional knee reconstruction from two-dimensional imaging has advanced substantially and shows strong translational potential for selected use cases, although this potential must be weighed against the methodological limitations of the current evidence base. Across the 28 included studies, contemporary methods using multi-view calibrated radiography or fluoroscopy commonly report surface errors on the order of approximately 0.8–1.5 mm, with several systems achieving ~0.5–1.0 mm root mean square or mean point-to-surface distance under controlled conditions relative to volumetric reference models (CT, MRI, or EOS-based reconstructions). Processing times for these approaches range from sub-second to a few minutes, which is compatible with many preoperative and intraoperative workflows. Reported radiation exposure for biplanar radiography is substantially lower than for CT, often by about an order of magnitude, which supports applications requiring serial imaging or use in younger patients. Within this accuracy band, statistical shape models, deep learning, and hybrid frameworks converge toward similar performance in well-calibrated multi-view scenarios, differing mainly in data requirements, interpretability, and degree of automation.

Practical deployment now depends less on incremental accuracy gains and more on validation breadth and system integration. Priority needs include publicly available benchmark datasets with paired radiographs and volumetric ground truth spanning diverse pathologies and demographics; multi-center evaluations using consistent acquisition protocols; and standardized reporting of reconstruction error and kinematic precision using clearly defined, comparable metrics. Uncertainty quantification that can be surfaced to clinicians at the point of care, and explicit reporting of failure modes, are also essential. Interoperability with hospital information systems, calibration workflows that tolerate routine clinical variation, and user interfaces that minimize interaction time will determine whether these methods can be embedded into real-world clinical pathways. Regulatory classification, documentation suitable for software-as-medical-device review, and viable reimbursement models for software-driven reconstruction will strongly influence adoption.

Clinical value is most evident where reduced dose and cost can partially or fully substitute for CT, including preoperative planning when cross-sectional imaging is unavailable or undesirable, intraoperative updates when additional CT is impractical, and longitudinal monitoring of joint shape and alignment. Pediatric imaging and resource-limited settings stand to benefit from lower radiation and wider availability of radiography equipment. Methods that function with limited views, simplified models, or relaxed calibration requirements can extend access where multi-view acquisition or precise calibration is constrained. Demonstrations that link reconstruction outputs to patient-relevant outcomes, such as implant alignment, operative efficiency, complication rates, and revision risk will be decisive for widespread clinical acceptance.

The technical feasibility of 2D-to-3D knee reconstruction has been demonstrated to a level suitable for many orthopedic tasks, but the overall evidence base remains constrained by small and heterogeneous cohorts, limited representation of severe pathology, and frequent reliance on simulated images or DRR-based validation. The next phase requires a coordinated effort to establish shared benchmarks, conduct prospective multi-center studies, quantify and communicate uncertainty, integrate with clinical infrastructure, and document economic impact. With these elements in place, 2D-to-3D reconstruction can provide a lower-dose, cost-conscious complement or alternative to CT for clearly defined indications in knee surgery and assessment.

## Supporting information

S1 Appendix Adapted QUADAS-2 quality-assessment framework. Rationale for adaptation, domain definitions and signalling questions, the rating rubric, and the evidence-grounding statement for the 28 included studies.
(DOCX)

S2 Checklist PRISMA 2020 checklist. (DOCX)
