# Supplementary material for: Two-dimensional to three-dimensional knee reconstruction from radiographs and fluoroscopy: A systematic review of methods and accuracy (1995–2025)
Source: PLoS One. 2026 Aug 26;21(8):e0356987. doi: 10.1371/journal.pone.0356987 (PMC13513995; doi:10.1371/journal.pone.0356987)
Supplement: Supplementary file 1 [file pone.0356987.s001.docx]

**Adapted QUADAS-2 Quality Assessment Framework**

**For technical accuracy-validation studies of 2D-to-3D knee reconstruction**

*Companion to: "Two-Dimensional to Three-Dimensional Knee Reconstruction from

Radiographs and Fluoroscopy: A Systematic Review of Methods and Accuracy

(1995-2025)." Applied to all 28 included studies.*

---

**1. Rationale for adaptation**

QUADAS-2 (Whiting et al., Ann Intern Med 2011) is the standard tool for

appraising diagnostic accuracy studies. The included studies are not classical

diagnostic-accuracy studies (a binary index test versus a clinical reference)

but **technical accuracy-validation studies**: a computational reconstruction

method (the "index test") produces a 3D bone surface that is compared, on a

continuous metric (surface distance / RMSE / Hausdorff), against a volumetric

reference model (the "reference standard"). Both reviewers agreed that ROBINS-I,

RoB 2, and GRADE are inappropriate here because there is no intervention, no

clinical outcome, and no randomized comparison. QUADAS-2's four-domain

architecture, however, maps cleanly onto this setting once each domain's

signalling questions are reinterpreted. We therefore retain QUADAS-2's four

risk-of-bias domains and three applicability domains, and re-specify the

signalling questions and the Low/High/Unclear rubric for the reconstruction

context. This preserves comparability with the wider systematic-review

literature while respecting the technical nature of the evidence.

Two adaptations recur throughout and deserve emphasis up front:

- **DRR / simulated-from-CT validation is a circularity hazard.** Many methods

are evaluated on digitally reconstructed radiographs (DRRs) rendered from the

*same* CT volume that also provides the reference surface. The input and the

reference then share their origin, so the reported error measures

self-consistency of the rendering-and-fitting pipeline rather than real-world

reconstruction accuracy. We treat DRR-only validation as a **High** concern

for D3 (reference standard, circularity) and for applicability D1 (the inputs

are not real clinical radiographs).

- **Weak or "fuzzy" reference standards.** CT segmentation is the accepted

volumetric gold standard. References derived from MRI, EOS/SterEOS

reconstructions, or image-based CT-projection metrics are weaker surrogates;

where a study's primary reference is one of these (or where the study itself

reports a much larger error against true CT than against its fuzzy reference),

we raise D3 and applicability-D3 concern accordingly.

---

**2. Domain definitions and signalling questions**

**D1 — Patient / Sample Selection (risk of bias)**

*Could the selection of specimens/subjects have introduced bias?*

- SQ1. Was the sample representative of the intended clinical target (in-vivo

patients), rather than exclusively cadavers, anthropomorphic phantoms, or

DRR/simulated images?

- SQ2. Were subjects enrolled consecutively or at random, rather than by

convenience?

- SQ3. Was pathology (e.g. osteoarthritis, deformity, TKA candidacy)

represented, rather than healthy/asymptomatic anatomy only?

- SQ4. Was the *test* sample size adequate to support the accuracy claim

(guideline: n<10 test specimens or a single-sex/single-ethnicity training

base is a concern)?

**Rubric.** *Low* = in-vivo (or mixed) sample, adequate n, some pathology, clear

enrolment. *High* = cadaver/phantom-only, or healthy-only where a clinical claim

is made, or very small test n (<~10), or a narrowly restricted population.

*Unclear* = sample type or size not reported in retrievable text.

**D2 — Index Test: the reconstruction method (risk of bias)**

*Could conduct/interpretation of the reconstruction have introduced bias?*

- SQ1. Was the method applied/evaluated without using knowledge of the reference

surface to tune the result (no fitting to the test target)?

- SQ2. Were reconstruction parameters, modes, and thresholds prespecified rather

than tuned on the test data?

- SQ3. For SSM/DL methods, was the training set disjoint from the test set (no

leakage; leave-one-out or an explicit held-out split)?

- SQ4. Is the method sufficiently described to permit the above judgments (a

proprietary black box lowers transparency)?

**Rubric.** *Low* = prespecified pipeline, explicit train/test separation,

reproducible description. *High* = demonstrable tuning on test data or

train/test leakage. *Unclear* = proprietary/black-box method or insufficient

description to judge leakage/prespecification.

**D3 — Reference Standard (risk of bias)**

*Is the reference a valid, independent gold standard?*

- SQ1. Is the reference a true volumetric gold standard (independent CT

segmentation), rather than a weaker surrogate (MRI, EOS/SterEOS, image-only

metrics)?

- SQ2. Is the reference independent of the reconstruction inputs — in

particular, are inputs **real radiographs** rather than DRRs rendered from the

same CT that supplies the reference (circularity)?

- SQ3. Was the reference segmented/derived independently of the index result?

**Rubric.** *Low* = independent CT-segmentation reference with real image

inputs. *High* = DRR-from-CT circular validation, or a fuzzy/weaker reference

(MRI/SterEOS/image-metric) used as the primary standard. *Unclear* = reference

modality or independence not reported.

**D4 — Flow & Timing (risk of bias)**

*Did every specimen receive the same reference and enter the analysis?*

- SQ1. Did all specimens/subjects receive the same reference standard?
- SQ2. Were all enrolled specimens included in the accuracy analysis (exclusions

explained)?

- SQ3. Was the interval between the 2D acquisition and the reference acquisition

appropriate (no anatomy change; consistent pose handling)?

**Rubric.** *Low* = uniform reference, full inclusion, appropriate interval.

*High* = unexplained exclusions or inconsistent/again-inappropriate reference

timing. *Unclear* = flow/exclusions not reported.

**Applicability concerns (A1–A3)**

Rated Low/High/Unclear against the review question — clinically usable 3D knee

reconstruction from routine 2D imaging.

- **A1 Patient selection:** Do the specimens and images match real clinical use?

DRR/phantom/cadaver inputs or healthy-only cohorts raise concern.

- **A2 Index test:** Does the method as implemented match a deployable clinical

workflow (view count, calibration, automation)? (Rarely a concern here — most

methods target the clinical modality directly.)

- **A3 Reference standard:** Does the reference define the target anatomy as

clinical practice would? A non-CT (MRI/SterEOS/image-metric) reference raises

concern.

---

**3. Evidence base and grounding**

Ratings were grounded in the primary literature wherever retrievable. Of 28

studies: **13 were rated from retrieved full text**, **11 from the published

abstract **(plus the review's extracted metrics), and** 4 from the manuscript's

secondary description only** — flagged in the table as "rated from secondary

description — author verification needed" (Fleute & Lavallée 1999; Gothard &

Anton 2025; Ha et al. 2024; and Hung-Chun Lee 2005, a Chinese-language Master's

thesis with no DOI and error reported in voxels rather than mm). DOIs were

resolved via Crossref/PubMed and are recorded in the retrieval log.

---

**4. Rating summary (counts across 28 studies)**

| Domain | Low | High | Unclear |

|---|---|---|---|

| D1 Patient/sample selection | 7 | 13 | 8 |

| D2 Index test (reconstruction) | 21 | 0 | 7 |

| D3 Reference standard | 16 | 9 | 3 |

| D4 Flow & timing | 18 | 0 | 10 |

| Applicability: patient selection | 10 | 14 | 4 |

| Applicability: index test | 26 | 1 | 1 |

| Applicability: reference standard | 16 | 9 | 3 |

Per-study ratings with one-line rationales, retrieval status, and resolved DOIs for all 28 studies are provided in the per-study ratings table in the main manuscript (Table 3) and summarised in the traffic-light figure (Fig 4); the domain-count summary appears in the table above.
