# Supplementary material for: Two-dimensional to three-dimensional knee reconstruction from radiographs and fluoroscopy: A systematic review of methods and accuracy (1995–2025)
Source: PLoS One. 2026 Aug 26;21(8):e0356987. doi: 10.1371/journal.pone.0356987 (PMC13513995; doi:10.1371/journal.pone.0356987)
Supplement: Supplementary file 3 [file pone.0356987.s003.docx]

**RESPONSE TO EDITOR’S COMMENTS**

Dear Editors,

We would like to thank the PLOS ONE editorial staff for handling our manuscript and for providing constructive feedback. We have made the necessary revisions to strengthen the manuscript, and we believe that the changes we implemented in accordance with the Editors’ recommendation have substantially improved the quality and scientific rigor of the study.

Please find below our detailed response to the reviewer comment, followed by explanations of revisions made within the text.

**Reviewer Comment (Comment #1)**

*“The manuscript imposed language restrictions during the literature search (English-only), without adequate justification. Excluding studies published in other languages can increase the risk of bias. We have concerns about the comprehensiveness of the review and therefore cannot consider it for publication unless this restriction is addressed.”*

**Answer to Comment #1**

We sincerely appreciate this important observation and fully agree with the reviewer’s concern regarding language-based exclusion bias. In response to this comment, we have completed the following updates:

1. Removed all English-language restrictions from the search strategy across all databases.
2. Conducted a new comprehensive multilingual search, which included screening results in Mandarin, Spanish, French, Portuguese, Korean, Russian, etc., We used translation tools and native-speaker consultation, in the same manner as we originally did for English.
3. Identified and included one newly eligible study (published in Mandarin) that satisfies all other inclusion criteria and was integrated into both the narrative review and quantitative synthesis.
4. Updated all PRISMA screening numbers and study totals, increasing the number of included studies from 27 to 28.
5. Revised the Methods section, removing language limitations and adding clarification detailing that English language is no longer an exclusion criteria (page 7).
6. Revised the Results and Discussion sections to reflect the expanded dataset and updated performance metrics.
7. Updated related figures, tables, and synthesis wording to incorporate the additional work and maintain internal consistency.

As a result, these revisions can be found in Methods: Databases and Search Strategy, Methods: Eligibility Criteria, Results: Overview of Included Studies, and the PRISMA Flow Diagram, visible in Track Changes version on pages 2–25.

We thank the reviewer again for highlighting this important point. We are confident that these changes substantially improve the comprehensiveness, scientific validity, and global relevance of the review.

As authors, we confirm that the manuscript now adheres fully to the PLOS ONE preparation guidelines. We respectfully request reconsideration of our manuscript for publication.
