# Supplementary material for: Two-dimensional to three-dimensional knee reconstruction from radiographs and fluoroscopy: A systematic review of methods and accuracy (1995–2025)
Source: PLoS One. 2026 Aug 26;21(8):e0356987. doi: 10.1371/journal.pone.0356987 (PMC13513995; doi:10.1371/journal.pone.0356987)
Supplement: Supplementary file 4 [file pone.0356987.s004.docx]

**Response to Reviewers**

**Manuscript PONE-D-25-62662**

*Two-Dimensional to Three-Dimensional Knee Reconstruction from Radiographs and Fluoroscopy: A Systematic Review of Methods and Accuracy (1995–2025)*

Dear Dr. Khasawneh and Reviewers,

We thank the Academic Editor and both reviewers for their careful and constructive evaluation of our manuscript. The comments have materially strengthened the work. In direct response, we have added a formal quality assessment (an adapted QUADAS-2 applied to all 28 included studies), added predefined quantitative criteria for our translational-readiness classification and softened the associated language, added an explanatory paragraph on the non-comparability of accuracy metrics, expanded the discussion of validation heterogeneity, improved the reproducibility of the search description, split the former Table 1 into two tables, regenerated Figure 3 for legibility, and reconciled every internal-consistency issue that was identified. We also identified and corrected an omission in the reference list (entries 31–48 were missing from the bibliography) and a duplicated reference number in the former Table 1.

Below we respond to each point in turn. Reviewer and editor comments are shown in italics; our responses follow. Page and section references correspond to the revised manuscript with tracked changes.

**Academic Editor Comments**

**Editor Comment 1 — Terminology (metrics)**

*Throughout the manuscript, RMSE, RMS, P2S, surface error, Hausdorff distance, and Dice score are occasionally discussed together. Although the manuscript notes their heterogeneity, readers may mistakenly compare these metrics directly. A short explanatory paragraph or footnote explaining differences between these metrics would improve clarity.*

We agree. We have added an explanatory paragraph to the Methods (Data Synthesis subsection) that defines each metric and explains why they are not interchangeable: RMSE/RMS summarize average squared deviation and are sensitive to large local errors; point-to-surface (P2S)/mean surface distance measure average nearest-point distance without requiring point correspondence; the 95th-percentile Hausdorff distance (HD95) is a near-worst-case boundary deviation and is systematically larger than a mean surface distance for the same reconstruction; and the Dice coefficient is a dimensionless overlap ratio (0–1) that cannot be converted to a millimetre error. We state explicitly that all reported values are presented with their original metric and units and that no direct numerical ranking across metrics is implied. This point is reinforced in the caption of the new performance-metrics table (Table 2).

**Editor Comment 2 — Search cutoff**

*The search ended in November 2025. Please confirm that no additional studies published before manuscript submission were omitted.*

We confirm that all database, register, and grey-literature searches were completed immediately prior to the initial submission and that no eligible study published before the submission date was knowingly omitted. We have added this explicit confirmation to the Methods (Databases and Search Strategy). In the course of this check we also noted and corrected an inconsistency in the front-matter date: the manuscript now records the submission date as November 25, 2025, so that the last search update (November 20, 2025) correctly precedes submission. The multilingual search added during the previous revision did not identify additional English-language studies beyond those already captured.

**Editor Comment 3 — Figures**

*Figure 3 is informative but visually dense. Increasing font size and simplifying labels would improve readability.*

Figure 3 has been regenerated. The three-panel dot/stacked/grouped layout has been simplified to two clearly labelled panels — (A) absolute study counts by method family and period, and (B) relative prevalence within each period — with substantially larger fonts, a single shared legend, direct value labels on the bars, and simplified family names. Critically, the panel counts are now locked to the values reported in the text (see Reviewer 1, Comment 3): 15 statistical shape models, 8 deep learning, 2 parametric, and 3 hybrid across the three periods (n = 3, 8, and 17).

**Editor Comment 4 — Tables**

*Table 1 contains valuable information but is difficult to read. Consider splitting it into two tables: Technical characteristics and Performance metrics.*

Done. The former Table 1 has been split into Table 1 (Technical characteristics: bone/region, algorithm family, core technique, input modality, and number of views) and Table 2 (Performance metrics: reported accuracy, accuracy-metric type, and stated validation reference). Splitting the columns also made room to add an explicit accuracy-metric-type column and a validation-reference column, which support the metric-comparability point (Editor Comment 1) and the validation-heterogeneity discussion (Reviewer 2).

**Reviewer 1**

**Reviewer 1, Comment 1 — PROSPERO registration**

*The authors say they did not register the protocol in PROSPERO because the search was iterative. I don’t find that convincing — an iterative search doesn’t prevent you from registering a protocol, and for a systematic review I’d expect either a registration or, at minimum, an honest acknowledgment of its absence as a limitation.*

We accept this. We have removed the “iterative search” justification and now state plainly in the Methods that the protocol was not prospectively registered in PROSPERO, and acknowledge this as a limitation — noting that prospective registration improves transparency and reduces the risk of selective reporting, and that an iterative search does not preclude registration. We describe the mitigating steps we did take (an a priori operating-procedure document, and full internal documentation of search strings, dates, eligibility criteria, and protocol deviations, all reported here).

**Reviewer 1, Comment 2 — Structured risk-of-bias / QUADAS-2**

*There’s no structured risk-of-bias assessment. … the studies they include are essentially accuracy-validation studies against a volumetric reference, which is exactly what QUADAS-2 was designed for. I’d ask them to apply an adapted QUADAS-2 across the included studies and summarize it.*

We agree, and this was also Reviewer 2’s primary concern. We have applied an adapted QUADAS-2 to all 28 included studies. We retained QUADAS-2’s four risk-of-bias domains (patient/sample selection, index test, reference standard, flow and timing) and three applicability domains, and rewrote the signalling questions for the reconstruction context. Two adaptations are central: validation on digitally reconstructed radiographs (DRRs) rendered from the same CT that supplies the reference is treated as a high risk of bias for the reference-standard domain (circularity), and non-CT references (MRI, EOS/SterEOS, image-only metrics) are treated as weaker standards. To ground the ratings we retrieved the primary literature: judgments rest on full text for 13/28 studies, on the published abstract for 11/28, and, for four studies whose full text could not be obtained, on the review’s extracted description (these rows are flagged for author verification). The full framework and rubric are provided as supporting information (S1 Appendix); domain-level results appear in the new Figure 4 (traffic-light summary) and per-study ratings in the new Table 3, with a summary narrative added to the Results/Discussion. In brief, risk of bias concentrates in patient/sample selection (13/28 high) and the reference standard (9/28 high), while the reconstruction methods themselves (index test: 21/28 low, no high) and analysis flow (18/28 low, no high) are generally sound — i.e., the methods are well built, but the evidence for real-world clinical accuracy is limited by how they were validated.

**Reviewer 1, Comment 3 — Internal inconsistencies**

*The initial pool reported in the text (1,254) doesn’t line up cleanly with the PRISMA flow diagram. The method-family counts in the text don’t match Figure 3 … The deep-learning share for the most recent period is 41% in the figure caption and 47% in the figure. And in Table 1 the same reference number appears twice (Li et al., 2014 and Baka et al., 2014). … together they need a careful pass to reconcile.*

We have reconciled each of these:

- Initial pool. The text figure of “1,254” was incorrect and did not match the PRISMA diagram. The correct total is 1,268 records identified (1,160 from databases, 14 from registers, and 94 from other sources); after removing 342 duplicates and 28 records excluded by automation tools, 804 records were screened, and 28 studies were included. The text now matches Figure 1 exactly, and the garbled sentence that implied 28 studies remained “after duplicate removal” has been rewritten.
- Method-family counts. The text counts (15 SSM / 8 DL / 2 parametric / 3 hybrid = 28) were correct; the previous Figure 3 used a different grouping. Figure 3 has been regenerated on the same family scheme as the text, so figure and text now agree (period totals n = 3, 8, 17).
- Deep-learning share. The 41% value was a mislabelling: in the most recent period (2016–2025), deep learning/AI accounts for 47% (8/17) of studies, while 41% (7/17) is the statistical-shape-model share. The regenerated Figure 3 and its caption now report 47% for deep learning and identify 41% as the SSM share, resolving the 41%/47% discrepancy.
- Duplicated reference number. In the former Table 1, Li et al. (2014) was mislabelled as [33], the number correctly used for Baka et al. (2014). The body text already distinguished them correctly (Baka 2014 = [33]; Li 2014 = [34]); we have corrected the table entry to [34]. While verifying this we found that the bibliography had been truncated at entry [30], so references [31]–[48] were missing entirely; we have reconstructed all of these entries from the primary sources (DOIs verified via Crossref) and added them to the reference list.

**Reviewer 1, Comment 4 — Framing of “clinical readiness”**

*I’d gently push back on the framing of “clinical readiness.” … describing some methods as “ready for deployment” feels stronger than the evidence supports. I’d prefer to see the translational potential framed against those limitations.*

We have softened this framing throughout. The Abstract and Conclusion no longer state that the field has “progressed to clinical readiness”; they now describe strong translational potential weighed explicitly against the methodological limitations (small validation cohorts, limited pathological representation, and frequent reliance on simulated/DRR validation). The former “Methods ready for deployment” tier has been renamed “Deployment-candidate methods,” and we now state that meeting the accuracy and speed bar is necessary but not sufficient for deployment — each such method was validated in a small cohort and none has undergone the multi-centre, prospective, pathology-spanning evaluation that clinical deployment would require. This reframing is consistent with the QUADAS-2 findings added in response to Comment 2.

**Reviewer 2**

**Reviewer 2, Comment 1 — Lack of formal quality assessment**

*This is the most important limitation. … Although conventional tools such as ROBINS-I may not be appropriate, there are alternative approaches for assessing methodological quality of diagnostic or AI studies. Without any structured quality assessment, readers cannot judge the reliability of the included evidence.*

We fully agree, and have addressed this with the adapted QUADAS-2 assessment described in our response to Reviewer 1, Comment 2. All 28 studies are now appraised across four risk-of-bias domains and three applicability domains against a prespecified rubric, grounded in the retrieved primary literature. The framework and rationale are provided in S1 Appendix, the domain-level distribution in Figure 4, and per-study ratings in Table 3, with an interpretive narrative in the Results/Discussion. This gives readers an explicit, reproducible basis for judging the reliability of the evidence.

**Reviewer 2, Comment 2 — Clinical-readiness classification appears subjective**

*The manuscript divides methods into Ready for deployment / Near-ready / Research phase. However, these categories appear to be based on the authors’ own interpretation. No predefined criteria are provided.*

We have added explicit, predefined quantitative criteria for the three tiers, now presented in the new Table 4 and stated a priori in the text. In brief: Deployment-candidate = accuracy <1 mm, processing <1 min, substantially automated, validated on real (non-simulated) images; Near-ready = accuracy 1–2 mm, processing <5 min, at most limited manual interaction; Research-phase = accuracy >2 mm or processing >5 min, extensive manual interaction, or simulated/DRR-only validation. A method is assigned to a tier only if it meets all criteria for that tier, and to the lower tier where criteria span tiers. We also state that these thresholds are pragmatic (derived from reported orthopaedic surgical-planning tolerances) and are intended as a transparent classification rather than a claim of regulatory or clinical qualification.

**Reviewer 2, Comment 3 — No quantitative synthesis**

*The authors correctly explain why a meta-analysis was not feasible.*

We thank the reviewer for acknowledging this. We have retained the narrative-synthesis rationale, and the new metric-terminology paragraph (Editor Comment 1) further clarifies why pooling across heterogeneous metrics would be inappropriate. No change beyond this clarification was required.

**Reviewer 2, Comment 4 — Validation heterogeneity deserves deeper discussion**

*The manuscript briefly mentions that some studies used CT, MRI, EOS, DRRs. However, these validation strategies are not equivalent.*

We have added a dedicated discussion of validation heterogeneity to the Limitations, explaining why the reference standards are not equivalent and cannot be pooled at face value: CT segmentation provides genuine volumetric ground truth; MRI-derived models depend on soft-tissue-optimised contrast and segmentation choices that shift the apparent bone boundary; EOS/SterEOS reconstructions are themselves model-based estimates (a “fuzzy” reference rather than an independent gold standard); and DRRs generated from the same CT used to build or evaluate the model introduce circularity. We illustrate the effect with Babazadeh et al. (2025), who reported 0.88 mm against a SterEOS reference but 2.70 mm against true CT. We now tag studies by reference-standard type (Table 2) so accuracies are read within, not across, validation designs, and this distinction is formalized in the QUADAS-2 reference-standard domain.

**Reviewer 2, Comment 5 — Search reproducibility (Google Scholar)**

*The search strategy is well described. However, Google Scholar searches are difficult to reproduce. Please specify: exact search strings, query order, date of each search, and how duplicates across searches were handled.*

We have expanded the Methods to make the Google Scholar and arXiv searches reproducible. We now give the exact ordered set of six sub-queries, note that they were run without date restriction with the first 500 results of each screened in the default relevance ranking, state that each sub-query was run on the same dated search and its result count logged, and describe duplicate handling: records from Google Scholar and arXiv were exported and de-duplicated against the database and register results in the reference manager using DOI and title matching before screening, so no record was screened or counted twice across sources.

We believe these revisions substantially strengthen the rigour and transparency of the review and fully address the points raised. We thank the editor and reviewers again for their time and constructive input.
