# Supplementary material for: Two-dimensional to three-dimensional knee reconstruction from radiographs and fluoroscopy: A systematic review of methods and accuracy (1995–2025)
Source: PLoS One. 2026 Aug 26;21(8):e0356987. doi: 10.1371/journal.pone.0356987 (PMC13513995; doi:10.1371/journal.pone.0356987)
Supplement: Peer review history

# Author comment: Author response to Decision Letter 0

**DOI:** 10.1371/journal.pone.0356987.r001

26 Nov 2025

Attachment Submitted filename: Rebuttal Letter.docx

# Peer review: Decision Letter 0

**DOI:** 10.1371/journal.pone.0356987.r002

**Authors:** Ramada Khasawneh

8 Jul 2026

Dear Dr. Travascio,

Thank you for submitting your manuscript to PLOS ONE. After careful consideration, we feel that it has merit but does not fully meet PLOS ONE’s publication criteria as it currently stands. Therefore, we invite you to submit a revised version of the manuscript that addresses the points raised during the review process.

==============================

Terminology --> Throughout the manuscript,  RMSE, RMS, P2S, surface error, Hausdorff distance, and Dice score are occasionally discussed together.

Although the manuscript notes their heterogeneity, readers may mistakenly compare these metrics directly.

A short explanatory paragraph or footnote explaining differences between these metrics would improve clarity.

Search cutoff-->  The search ended in November 2025.

Please confirm that no additional studies published before manuscript submission were omitted.

Figures -->  Figure 3 is informative but visually dense.

Increasing font size and simplifying labels would improve readability.

Tables -->  Table 1 contains valuable information but is difficult to read.

Consider splitting it into two tables: Technical characteristics and Performance metrics

==============================

Please submit your revised manuscript by Aug 22 2026 11:59PM. If you will need more time than this to complete your revisions, please reply to this message or contact the journal office at plosone@plos.org. When you're ready to submit your revision, log on to https://www.editorialmanager.com/pone/ and select the 'Submissions Needing Revision' folder to locate your manuscript file.

- A letter that responds to each point raised by the academic editor and reviewer(s). You should upload this letter as a separate file labeled 'Response to Reviewers'.
- A marked-up copy of your manuscript that highlights changes made to the original version. You should upload this as a separate file labeled 'Revised Manuscript with Track Changes'.
- An unmarked version of your revised paper without tracked changes. You should upload this as a separate file labeled 'Manuscript'.

If you would like to make changes to your financial disclosure, please include your updated statement in your cover letter. Guidelines for resubmitting your figure files are available below the reviewer comments at the end of this letter.

If applicable, we recommend that you deposit your laboratory protocols in protocols.io to enhance the reproducibility of your results. Protocols.io assigns your protocol its own identifier (DOI) so that it can be cited independently in the future. For instructions see: https://journals.plos.org/plosone/s/submission-guidelines#loc-laboratory-protocols. Additionally, PLOS ONE offers an option for publishing peer-reviewed Lab Protocol articles, which describe protocols hosted on protocols.io. Read more information on sharing protocols at https://plos.org/protocols?utm_medium=editorial-email&utm_source=authorletters&utm_campaign=protocols.

As the corresponding author, your ORCID iD is verified in the submission system and will appear in the published article. PLOS supports the use of ORCID, and we encourage all coauthors to register for an ORCID iD and use it as well. Please encourage your coauthors to verify their ORCID iD within the submission system before final acceptance, as unverified ORCID iDs will not appear in the published article. *Only*  the individual author can complete the verification step; PLOS staff *cannot*  verify ORCID iDs on behalf of authors.

We look forward to receiving your revised manuscript.

Kind regards,

Ramada Rateb Khasawneh

Academic Editor

PLOS One

Journal Requirements:

When submitting your revision, we need you to address these additional requirements.

1. Please ensure that your manuscript meets PLOS ONE's style requirements, including those for file naming. The PLOS ONE style templates can be found at

https://journals.plos.org/plosone/s/file?id=wjVg/PLOSOne_formatting_sample_main_body.pdf and

https://journals.plos.org/plosone/s/file?id=ba62/PLOSOne_formatting_sample_title_authors_affiliations.pdf

2. If the reviewer comments include a recommendation to cite specific previously published works, please review and evaluate these publications to determine whether they are relevant and should be cited. There is no requirement to cite these works unless the editor has indicated otherwise.

Additional Editor Comments:

Terminology --> Throughout the manuscript, RMSE, RMS, P2S, surface error, Hausdorff distance, and Dice score are occasionally discussed together.

Although the manuscript notes their heterogeneity, readers may mistakenly compare these metrics directly.

A short explanatory paragraph or footnote explaining differences between these metrics would improve clarity.

Search cutoff--> The search ended in November 2025.

Please confirm that no additional studies published before manuscript submission were omitted.

Figures --> Figure 3 is informative but visually dense.

Increasing font size and simplifying labels would improve readability.

Tables --> Table 1 contains valuable information but is difficult to read.

Consider splitting it into two tables: Technical characteristics and Performance metrics

[Note: HTML markup is below. Please do not edit.]

Reviewers' comments:

Reviewer's Responses to Questions

**Comments to the Author**

1. Is the manuscript technically sound, and do the data support the conclusions?

Reviewer #1: Partly

Reviewer #2: Yes

**********

2. Has the statistical analysis been performed appropriately and rigorously?

Reviewer #1: N/A

Reviewer #2: Yes

**********

3. Have the authors made all data underlying the findings in their manuscript fully available?

The PLOS Data policy

Reviewer #1: Yes

Reviewer #2: Yes

**********

4. Is the manuscript presented in an intelligible fashion and written in standard English?

Reviewer #1: Yes

Reviewer #2: Yes

**********

Reviewer #1: I read this manuscript with interest. It tackles a genuinely useful question — whether 2D-to-3D knee reconstruction from radiographs and fluoroscopy has matured enough to stand in for CT — and it pulls together three decades of work that has never really been synthesized in one place for the knee specifically. The writing is clear, the search is broad, and the narrative synthesis is a sensible choice given how heterogeneous the accuracy metrics and validation designs are. I think this can become a solid contribution, but there are a few things I would want addressed before I’d be comfortable seeing it published.

My main concern is on the methodological side. The authors say they did not register the protocol in PROSPERO because the search was iterative. I don’t find that convincing — an iterative search doesn’t prevent you from registering a protocol, and for a systematic review I’d expect either a registration or, at minimum, an honest acknowledgment of its absence as a limitation. Related to this, there’s no structured risk-of-bias assessment. The authors explain why ROBINS-I, RoB 2, and GRADE don’t fit, and I agree with that reasoning, but the studies they include are essentially accuracy-validation studies against a volumetric reference, which is exactly what QUADAS-2 was designed for. I’d ask them to apply an adapted QUADAS-2 across the included studies and summarize it. As it stands, the review has no formal appraisal of study quality, and I don’t think that meets current expectations.

I also noticed several internal inconsistencies that made me less confident in the numbers. The initial pool reported in the text (1,254) doesn’t line up cleanly with the PRISMA flow diagram. The method-family counts in the text don’t match Figure 3 — the text gives 15 SSM / 8 DL / 2 parametric / 3 hybrid, but the figure adds up differently. The deep-learning share for the most recent period is 41% in the figure caption and 47% in the figure. And in Table 1 the same reference number appears twice (Li et al., 2014 and Baka et al., 2014). None of these are fatal, but together they need a careful pass to reconcile.

On the conclusions, I’d gently push back on the framing of “clinical readiness.” The authors themselves point out the recurring weaknesses — small cohorts, often under 50 subjects, limited pathological cases, and a lot of DRR or simulated validation — so describing some methods as “ready for deployment” feels stronger than the evidence supports. I’d prefer to see the translational potential framed against those limitations rather than as something already established.

Reviewer #2: This manuscript presents a comprehensive systematic review of 3D knee reconstruction from 2D radiographs and fluoroscopy. The topic is timely and clinically relevant given the increasing interest in reducing CT utilization while maintaining sufficient anatomical accuracy for orthopedic planning and navigation. The manuscript is generally well organized, covers approximately three decades of technological development, and provides a useful chronological overview of statistical shape models, model-based registration, deep learning, and hybrid techniques.

Lack of formal quality assessment --> This is the most important limitation.

The authors state that no formal risk-of-bias tool was applied because the included studies were technical developments.

Although conventional tools such as ROBINS-I may not be appropriate, there are alternative approaches for assessing methodological quality of diagnostic or AI studies.

Without any structured quality assessment, readers cannot judge the reliability of the included evidence.

Clinical readiness classification appears subjective

The manuscript divides methods into:

-Ready for deployment

-Near-ready

-Research phase

However, these categories appear to be based on the authors' own interpretation.

No predefined criteria are provided.

No quantitative synthesis --> The authors correctly explain why a meta-analysis was not feasible.

Validation heterogeneity deserves deeper discussion

The manuscript briefly mentions that some studies used

-CT

-MRI

-EOS

-DRRs

However, these validation strategies are not equivalent.

Methods --> The search strategy is well described.

However, Google Scholar searches are difficult to reproduce.

Please specify: exact search strings, query order, date of each search, and how duplicates across searches were handled.

**********

what does this mean?). If published, this will include your full peer review and any attached files.

If you choose “no”, your identity will remain anonymous but your review may still be made public.

**Do you want your identity to be public for this peer review?**  For information about this choice, including consent withdrawal, please see our Privacy Policy

Reviewer #1: No

Reviewer #2: No

**********

[NOTE: If reviewer comments were submitted as an attachment file, they will be attached to this email and accessible via the submission site. Please log into your account, locate the manuscript record, and check for the action link "View Attachments". If this link does not appear, there are no attachment files.]

To ensure your figures meet our technical requirements, please review our figure guidelines: https://journals.plos.org/plosone/s/figures

You may also use PLOS’s free figure tool, NAAS, to help you prepare publication quality figures: https://journals.plos.org/plosone/s/figures#loc-tools-for-figure-preparation.

NAAS will assess whether your figures meet our technical requirements by comparing each figure against our figure specifications.

# Author comment: Author response to Decision Letter 1

**DOI:** 10.1371/journal.pone.0356987.r003

22 Jul 2026

Reviewer 1

Reviewer 1, Comment 1 — PROSPERO registration

The authors say they did not register the protocol in PROSPERO because the search was iterative. I don’t find that convincing — an iterative search doesn’t prevent you from registering a protocol, and for a systematic review I’d expect either a registration or, at minimum, an honest acknowledgment of its absence as a limitation.

We accept this. We have removed the “iterative search” justification and now state plainly in the Methods that the protocol was not prospectively registered in PROSPERO, and acknowledge this as a limitation — noting that prospective registration improves transparency and reduces the risk of selective reporting, and that an iterative search does not preclude registration. We describe the mitigating steps we did take (an a priori operating-procedure document, and full internal documentation of search strings, dates, eligibility criteria, and protocol deviations, all reported here).

Reviewer 1, Comment 2 — Structured risk-of-bias / QUADAS-2

There’s no structured risk-of-bias assessment. … the studies they include are essentially accuracy-validation studies against a volumetric reference, which is exactly what QUADAS-2 was designed for. I’d ask them to apply an adapted QUADAS-2 across the included studies and summarize it.

We agree, and this was also Reviewer 2’s primary concern. We have applied an adapted QUADAS-2 to all 28 included studies. We retained QUADAS-2’s four risk-of-bias domains (patient/sample selection, index test, reference standard, flow and timing) and three applicability domains, and rewrote the signalling questions for the reconstruction context. Two adaptations are central: validation on digitally reconstructed radiographs (DRRs) rendered from the same CT that supplies the reference is treated as a high risk of bias for the reference-standard domain (circularity), and non-CT references (MRI, EOS/SterEOS, image-only metrics) are treated as weaker standards. To ground the ratings we retrieved the primary literature: judgments rest on full text for 13/28 studies, on the published abstract for 11/28, and, for four studies whose full text could not be obtained, on the review’s extracted description (these rows are flagged for author verification). The full framework and rubric are provided as supporting information (S1 Appendix); domain-level results appear in the new Figure 4 (traffic-light summary) and per-study ratings in the new Table 3, with a summary narrative added to the Results/Discussion. In brief, risk of bias concentrates in patient/sample selection (13/28 high) and the reference standard (9/28 high), while the reconstruction methods themselves (index test: 21/28 low, no high) and analysis flow (18/28 low, no high) are generally sound — i.e., the methods are well built, but the evidence for real-world clinical accuracy is limited by how they were validated.

Reviewer 1, Comment 3 — Internal inconsistencies

The initial pool reported in the text (1,254) doesn’t line up cleanly with the PRISMA flow diagram. The method-family counts in the text don’t match Figure 3 … The deep-learning share for the most recent period is 41% in the figure caption and 47% in the figure. And in Table 1 the same reference number appears twice (Li et al., 2014 and Baka et al., 2014). … together they need a careful pass to reconcile.

We have reconciled each of these:

• Initial pool. The text figure of “1,254” was incorrect and did not match the PRISMA diagram. The correct total is 1,268 records identified (1,160 from databases, 14 from registers, and 94 from other sources); after removing 342 duplicates and 28 records excluded by automation tools, 804 records were screened, and 28 studies were included. The text now matches Figure 1 exactly, and the garbled sentence that implied 28 studies remained “after duplicate removal” has been rewritten.

• Method-family counts. The text counts (15 SSM / 8 DL / 2 parametric / 3 hybrid = 28) were correct; the previous Figure 3 used a different grouping. Figure 3 has been regenerated on the same family scheme as the text, so figure and text now agree (period totals n = 3, 8, 17).

• Deep-learning share. The 41% value was a mislabelling: in the most recent period (2016–2025), deep learning/AI accounts for 47% (8/17) of studies, while 41% (7/17) is the statistical-shape-model share. The regenerated Figure 3 and its caption now report 47% for deep learning and identify 41% as the SSM share, resolving the 41%/47% discrepancy.

• Duplicated reference number. In the former Table 1, Li et al. (2014) was mislabelled as [33], the number correctly used for Baka et al. (2014). The body text already distinguished them correctly (Baka 2014 = [33]; Li 2014 = [34]); we have corrected the table entry to [34]. While verifying this we found that the bibliography had been truncated at entry [30], so references [31]–[48] were missing entirely; we have reconstructed all of these entries from the primary sources (DOIs verified via Crossref) and added them to the reference list.

Reviewer 1, Comment 4 — Framing of “clinical readiness”

I’d gently push back on the framing of “clinical readiness.” … describing some methods as “ready for deployment” feels stronger than the evidence supports. I’d prefer to see the translational potential framed against those limitations.

We have softened this framing throughout. The Abstract and Conclusion no longer state that the field has “progressed to clinical readiness”; they now describe strong translational potential weighed explicitly against the methodological limitations (small validation cohorts, limited pathological representation, and frequent reliance on simulated/DRR validation). The former “Methods ready for deployment” tier has been renamed “Deployment-candidate methods,” and we now state that meeting the accuracy and speed bar is necessary but not sufficient for deployment — each such method was validated in a small cohort and none has undergone the multi-centre, prospective, pathology-spanning evaluation that clinical deployment would require. This reframing is consistent with the QUADAS-2 findings added in response to Comment 2.

Reviewer 2

Reviewer 2, Comment 1 — Lack of formal quality assessment

This is the most important limitation. … Although conventional tools such as ROBINS-I may not be appropriate, there are alternative approaches for assessing methodological quality of diagnostic or AI studies. Without any structured quality assessment, readers cannot judge the reliability of the included evidence.

We fully agree, and have addressed this with the adapted QUADAS-2 assessment described in our response to Reviewer 1, Comment 2. All 28 studies are now appraised across four risk-of-bias domains and three applicability domains against a prespecified rubric, grounded in the retrieved primary literature. The framework and rationale are provided in S1 Appendix, the domain-level distribution in Figure 4, and per-study ratings in Table 3, with an interpretive narrative in the Results/Discussion. This gives readers an explicit, reproducible basis for judging the reliability of the evidence.

Reviewer 2, Comment 2 — Clinical-readiness classification appears subjective

The manuscript divides methods into Ready for deployment / Near-ready / Research phase. However, these categories appear to be based on the authors’ own interpretation. No predefined criteria are provided.

We have added explicit, predefined quantitative criteria for the three tiers, now presented in the new Table 4 and stated a priori in the text. In brief: Deployment-candidate = accuracy <1 mm, processing <1 min, substantially automated, validated on real (non-simulated) images; Near-ready = accuracy 1–2 mm, processing <5 min, at most limited manual interaction; Research-phase = accuracy >2 mm or processing >5 min, extensive manual interaction, or simulated/DRR-only validation. A method is assigned to a tier only if it meets all criteria for that tier, and to the lower tier where criteria span tiers. We also state that these thresholds are pragmatic (derived from reported orthopaedic surgical-planning tolerances) and are intended as a transparent classification rather than a claim of regulatory or clinical qualification.

Reviewer 2, Comment 3 — No quantitative synthesis

The authors correctly explain why a meta-analysis was not feasible.

We thank the reviewer for acknowledging this. We have retained the narrative-synthesis rationale, and the new metric-terminology paragraph (Editor Comment 1) further clarifies why pooling across heterogeneous metrics would be inappropriate. No change beyond this clarification was required.

Reviewer 2, Comment 4 — Validation heterogeneity deserves deeper discussion

The manuscript briefly mentions that some studies used CT, MRI, EOS, DRRs. However, these validation strategies are not equivalent.

We have added a dedicated discussion of validation heterogeneity to the Limitations, explaining why the reference standards are not equivalent and cannot be pooled at face value: CT segmentation provides genuine volumetric ground truth; MRI-derived models depend on soft-tissue-optimised contrast and segmentation choices that shift the apparent bone boundary; EOS/SterEOS reconstructions are themselves model-based estimates (a “fuzzy” reference rather than an independent gold standard); and DRRs generated from the same CT used to build or evaluate the model introduce circularity. We illustrate the effect with Babazadeh et al. (2025), who reported 0.88 mm against a SterEOS reference but 2.70 mm against true CT. We now tag studies by reference-standard type (Table 2) so accuracies are read within, not across, validation designs, and this distinction is formalized in the QUADAS-2 reference-standard domain.

Reviewer 2, Comment 5 — Search reproducibility (Google Scholar)

The search strategy is well described. However, Google Scholar searches are difficult to reproduce. Please specify: exact search strings, query order, date of each search, and how duplicates across searches were handled.

We have expanded the Methods to make the Google Scholar and arXiv searches reproducible. We now give the exact ordered set of six sub-queries, note that they were run without date restriction with the first 500 results of each screened in the default relevance ranking, state that each sub-query was run on the same dated search and its result count logged, and describe duplicate handling: records from Google Scholar and arXiv were exported and de-duplicated against the database and register results in the reference manager using DOI and title matching before screening, so no record was screened or counted twice across sources.

We believe these revisions substantially strengthen the rigour and transparency of the review and fully address the points raised. We thank the editor and reviewers again for their time and constructive input.

Attachment Submitted filename: Response_to_Reviewers.docx

# Peer review: Decision Letter 1

**DOI:** 10.1371/journal.pone.0356987.r004

**Authors:** Ramada Khasawneh

11 Aug 2026

Two-Dimensional to Three-Dimensional Knee Reconstruction from Radiographs and Fluoroscopy: A Systematic Review of Methods and Accuracy (1995–2025)

PONE-D-25-62662R1

Dear Dr. Travascio,

We’re pleased to inform you that your manuscript has been judged scientifically suitable for publication and will be formally accepted for publication once it meets all outstanding technical requirements.

Within one week, you’ll receive an e-mail detailing the required amendments. When these have been addressed, you’ll receive a formal acceptance letter and your manuscript will be scheduled for publication.

An invoice will be generated when your article is formally accepted. Please note, if your institution has a publishing partnership with PLOS and your article meets the relevant criteria, all or part of your publication costs will be covered. Please make sure your user information is up-to-date by logging into Editorial Manager at Editorial Manager® and clicking the ‘Update My Information' link at the top of the page. For questions related to billing, please contact billing support.

If your institution or institutions have a press office, please notify them about your upcoming paper to help maximize its impact. If they’ll be preparing press materials, please inform our press team as soon as possible -- no later than 48 hours after receiving the formal acceptance. Your manuscript will remain under strict press embargo until 2 pm Eastern Time on the date of publication. For more information, please contact onepress@plos.org.

Kind regards,

Ramada Rateb Khasawneh

Academic Editor

PLOS One

Additional Editor Comments (optional):

Good luck

Reviewers' comments:

Reviewer's Responses to Questions

**Comments to the Author**

Reviewer #1: All comments have been addressed

Reviewer #2: All comments have been addressed

**********

2. Is the manuscript technically sound, and do the data support the conclusions?

Reviewer #1: Yes

Reviewer #2: Yes

**********

3. Has the statistical analysis been performed appropriately and rigorously?

Reviewer #1: (No Response)

Reviewer #2: I Don't Know

**********

4. Have the authors made all data underlying the findings in their manuscript fully available?

The PLOS Data policy

Reviewer #1: Yes

Reviewer #2: Yes

**********

5. Is the manuscript presented in an intelligible fashion and written in standard English?

Reviewer #1: Yes

Reviewer #2: Yes

**********

Reviewer #1: Acceptance recommended, no further revision required. For the record: (1) absence of prospective PROSPERO registration — acknowledged as a limitation, but still precludes external verification that eligibility criteria weren’t adjusted post hoc; (2) part of the QUADAS-2 assessment (15 of 28 studies) relied on abstracts or secondary descriptions rather than full text, somewhat weakening the risk-of-bias tool itself; (3) small evidence base (n=28) — appropriate for this specialized field, but conclusions on “clinical readiness” should be read as provisional. None of these points require further author action.

Reviewer #2: Overall, yes. The revised manuscript addresses most of the major concerns, and in several cases addresses them very well. Good Luck

**********

what does this mean?). If published, this will include your full peer review and any attached files.

If you choose “no”, your identity will remain anonymous but your review may still be made public.

**Do you want your identity to be public for this peer review?**  For information about this choice, including consent withdrawal, please see our Privacy Policy

Reviewer #1: No

Reviewer #2: No

**********

# Editor report: Acceptance letter

**DOI:** 10.1371/journal.pone.0356987.r005

**Authors:** Ramada Khasawneh

PONE-D-25-62662R1

PLOS One

Dear Dr. Travascio,

I'm pleased to inform you that your manuscript has been deemed suitable for publication in PLOS One. Congratulations! Your manuscript is now being handed over to our production team.

At this stage, our production department will prepare your paper for publication. This includes ensuring the following:

* All references, tables, and figures are properly cited

* All relevant supporting information is included in the manuscript submission,

* There are no issues that prevent the paper from being properly typeset

You will receive further instructions from the production team, including instructions on how to review your proof when it is ready. Please keep in mind that we are working through a large volume of accepted articles, so please give us a few days to review your paper and let you know the next and final steps.

Lastly, if your institution or institutions have a press office, please let them know about your upcoming paper now to help maximize its impact. If they'll be preparing press materials, please inform our press team within the next 48 hours. Your manuscript will remain under strict press embargo until 2 pm Eastern Time on the date of publication. For more information, please contact onepress@plos.org.

You will receive an invoice from PLOS for your publication fee after your manuscript has reached the completed accept phase. If you receive an email requesting payment before acceptance or for any other service, this may be a phishing scheme. Learn how to identify phishing emails and protect your accounts at https://explore.plos.org/phishing.

If we can help with anything else, please email us at customercare@plos.org.

Thank you for submitting your work to PLOS One and supporting open access.

Kind regards,

PLOS ONE Editorial Office Staff

on behalf of

Dr. Ramada Rateb Khasawneh

Academic Editor

PLOS One
